# Tumor bud-derived CCL5 recruits fibroblasts and promotes colorectal cancer progression via CCR5-SLC25A24 signaling

**DOI:** 10.1186/s13046-022-02300-w

**Published:** 2022-03-03

**Authors:** Ling-Fang Gao, Yan Zhong, Ting Long, Xia Wang, Jia-Xian Zhu, Xiao-Yan Wang, Zhi-Yan Hu, Zu-Guo Li

**Affiliations:** 1 Department of Pathology, Shenzhen Hospital, Southern Medical University, Shenzhen, 518101 Guangdong China; 2 Department of Pathology, School of Basic Medical Sciences, Southern Medical University, Guangzhou, 510515 Guangdong China; 3 Department of Pathology, Nanfang Hospital, Southern Medical University, Guangzhou, 510515 Guangdong China

**Keywords:** Tumor budding, CCL5, Fibroblast, Colorectal cancer, CCR5, SLC25A24, Angiogenesis, Collagen synthesis

## Abstract

**Background:**

Tumor budding is included in the routine diagnosis of colorectal cancer (CRC) and is considered a tumor prognostic factor independent of TNM staging. This study aimed to identify the fibroblast-mediated effect of tumor bud-derived C–C chemokine ligand 5 (CCL5) on the tumor microenvironment (TME).

**Methods:**

Recruitment assays and a human cytokine array were used to detect the main cytokines that CRC tumor buds secrete to recruit fibroblasts. siRNA transfection and inhibitor treatment were used to investigate the role of fibroblast CCL5 receptors in fibroblast recruitment. Subsequently, transcriptome sequencing was performed to explore the molecular changes occurring in fibroblasts upon stimulation with CCL5. Finally, clinical specimens and orthotopic xenograft mouse models were studied to explore the contribution of CCL5 to angiogenesis and collagen synthesis.

**Results:**

Hematoxylin–eosin staining and immunochemistry revealed a higher number of fibroblasts at the invasive front of CRC tissue showing tumor budding than at sites without tumor budding. In vitro experiments demonstrated that CCL5 derived from tumor buds could recruit fibroblasts by acting on the CCR5 receptors on fibroblasts. Tumor bud-derived CCL5 could also positively regulate solute carrier family 25 member 24 (SLC25A24) expression in fibroblasts, potentially activating pAkt-pmTOR signaling. Moreover, CCL5 could increase the number of α-SMA^high^ CD90^high^ FAP^low^ fibroblasts and thus promote tumor angiogenesis by enhancing VEGFA expression and making fibroblasts transdifferentiate into vascular endothelial cells. Finally, the results also showed that CCL5 could promote collagen synthesis through fibroblasts, thus contributing to tumor progression.

**Conclusions:**

At the invasive front of CRC, tumor bud-derived CCL5 can recruit fibroblasts via CCR5-SLC25A24 signaling, further promoting angiogenesis and collagen synthesis via recruited fibroblasts, and eventually create a tumor-promoting microenvironment. Therefore, CCL5 may serve as a potential diagnostic marker and therapeutic target for tumor budding in CRC.

**Supplementary Information:**

The online version contains supplementary material available at 10.1186/s13046-022-02300-w.

## Background

The relationship between tumors and the tumor microenvironment (TME) is analogous to that between seeds and the soil. Studies are gradually focusing not only on the tumor alone but also on the TME. Cancer-associated fibroblasts (CAFs), an indispensable part of the tumor stroma, have attracted increasing attention in recent years [1–3]. Evidence from both clinical and basic studies has revealed a strong association between the number of CAFs and poor clinical outcomes in several types of cancer, including breast cancer [4], cervical cancer [5], lung cancer [6], cholangiocarcinoma [7], and colorectal cancer (CRC) [8, 9]. The crosstalk between fibroblasts and tumor cells can lead to fibroblast activation, subsequently resulting in tumor metastasis [8], treatment resistance [10], and immunosuppression [11]. However, the mechanism underlying the initial transformation of fibroblasts to a tumor-promoting state remains unclear.

Tumor budding in CRC is defined as a single tumor cell or a cell cluster of up to four tumor cells, assessed in one hotspot (in a field measuring 0.785 mm^2^) at the invasive front [12, 13]. Studies have indicated a close link between tumor budding and a distinctive immune-suppressing microenvironment that promotes tumor invasion in gastric adenocarcinoma [14], stage I lung adenocarcinoma [15], pancreatic cancer [16], and CRC [17]. Nevertheless, the crosstalk between fibroblasts and tumor buds, and the mechanism through which the crosstalk occurs remain unknown.

C–C chemokine ligand 5 (CCL5), also known as Regulated upon Activation, Normal T-cell Expressed, and Secreted (RANTES), interacts with the G-protein–coupled receptors CCR1, CCR3, CCR4, CCR5, GPR75, and CD44 [18, 19]. CCL5 is expressed in T lymphocytes, macrophages, platelets, synovial fibroblasts, the tubular epithelia, and tumor cells [20]. As a chemokine, CCL5 plays an active role in recruiting a variety of leukocytes to sites of inflammation. In CRC, macrophage-derived CCL5 facilitates the immune escape of cancer cells via the p65/STAT3-CSN5-PD-L1 pathway [21]. Moreover, tumor-derived CCL5 enhances TGF-β secretion in T regulatory cells via the CCL5/CCR5 axis, thereby blocking the killing function of CD8^+^ T cells [22]. Nevertheless, the biological effect of tumor-derived CCL5 on fibroblasts remains elusive. Moreover, fibroblasts have also been recognized as an important source of CCL5 [23–25], and whether human colorectal fibroblasts influence themselves through CCL5 autocrine function remains to be explored.

Here, we report for the first time that CCL5 secreted by CRC tumor buds at the invasive front can recruit surrounding fibroblasts through the CCR5-solute carrier family 25 member 24 (SLC25A24) pathway, and further promote CRC progression via fibroblast-mediated increases in angiogenesis and collagen synthesis. These findings show that CCL5 may serve as a potential diagnostic marker and therapeutic target for tumor budding in CRC.

## Methods

### Antibodies, small interfering RNA (siRNA), and primer sequences

The primary antibodies used in the study are summarized in Additional file 6: Table S1. The siRNA and primer sequences used in the study are summarized in Additional file 7: Table S2 and Additional file 8: Table S3 respectively.

### Cell lines and cell culture

The human CRC cell lines LS174T (CL-188), RKO (CRL-2577), DLD-1 (CCL-221), Caco2 (HTB-37), SW620 (CCL-227), HCT-8 (CCL-244), HCT116 (CCL-247), and HCT-15 (CCL-225); the human normal colorectal epithelial cell line FHC (CRL-1831); and the human normal colorectal fibroblast cell line CCD-18Co (CRL-1459) were all purchased from the American Type Culture Collection (ATCC). All human CRC cells were cultured in RPMI-1640 medium (Gibco, C11875500BT) supplemented with 10% fetal bovine serum (FBS) (ExCell Bio, FND500). FHC was cultured in RPMI-1640 medium supplemented with 15% FBS, and CCD-18Co was cultured in Eagle’s Minimum Essential Medium (EMEM) (ATCC, 30–2003) supplemented with 10% FBS. All cells were cultured at 37 °C in humidified atmosphere containing 5% CO_2_. Cell line certificates of analysis were obtained from the ATCC. All cell lines were negative for mycoplasma.

### Primary normal colorectal fibroblasts: extraction and culture

Fresh human normal colorectal mucosae were cut using surgical scissors and then enzymatically dissociated in a mixture of type IV collagenase (2.0 mg/ml, Sigma, C5138), hyaluronidase (0.4 mg/ml, Sigma, H1115000) and DNase (25 U/ml, Solarbio, D8071) at a constant temperature of 37 °C for 2 h. The tissues were then passed through a 40-μm cell strainer to generate a single-cell suspension. The cell suspensions were centrifuged at 300 × *g* for 12 min; the supernatant was discarded, and the cell pellet was resuspended in EMEM. Primary cells were then plated at a density of 1 × 10^5^ viable cells in 25 cm^2^ adherent flasks and cultured at 37 °C in EMEM with 10% FBS in humidified atmosphere containing 5% CO_2_ (enzyme digestion method). The tissues that could not pass through the strainer were transferred to 25 cm^2^ adherent flasks. EMEM supplemented with 10% FBS was added to the adherent flasks after 24 h, when the tissues had stuck to the bottom. The tissues were incubated until fibroblasts crawled out of them (improved tissue planting method).

### Clinical specimens

Clinical CRC specimens were obtained from patients who were pathologically diagnosed with CRC at Shenzhen Hospital, Southern Medical University. The study was approved by the ethics committee of Shenzhen Hospital, Southern Medical University, China.

### Identification and quantification of tumor budding

Tumor budding was identified based on the presence of a single tumor cell or a tumor cell cluster of up to 4 cells at the invasive front of CRC tumors. The quantification of tumor budding was performed according to five procedures proposed by the International Tumor Budding Consensus Conference (ITBCC) 2016 for reporting tumor budding in CRC during daily diagnostic practice [13].

### Immunohistochemistry (IHC)

IHC was performed using paraffin-embedded sections of human CRC tissue following the standard LSAB protocol (Dako). Primary antibodies against α-SMA, CD90, FAP, CCL5, SLC25A24, CD31, and VEGFA were used for IHC. The degree of staining was observed and scored independently by three pathologists. The percent positivity of CCL5 staining was scored from 0 to 4, as follows: 0 (< 5%), 1 (5–25%), 2 (26–50%), 3 (51–75%), and 4 (> 75%). The staining intensity was scored on a 4-point scale, as follows: 0 (no staining), 1 (weak staining, light yellow), 2 (moderate staining, yellowish brown), and 3 (strong staining, brown). Subsequently, the CCL5 expression score was calculated by multiplying the percent positivity score with the staining intensity score. Accordingly, the expression of CCL5 was classified as low (0–4), medium (5–8), or high (9–12).

### Immunofluorescence (IF) analysis of cells and CRC tissue

IF analysis of cells was performed as previously described [26]. For IF analysis of CRC tissue, the steps before primary antibody incubation were the same as those used for IHC. The steps after primary antibody incubation were the same as those used for IF analysis of cells. Images of cells were acquired using a laser scanning confocal microscope (Olympus, Japan), and images of CRC tissue were acquired using a fluorescence microscope (Olympus, Japan).

### Recruitment assay

Boyden transwell chambers (Corning, 353,097) were used according to manufacturer’s instructions. Briefly, 2 × 10^4^ fibroblasts were added to the upper chambers. The lower chamber contained of the following: 1 × 10^5^ human CRC tumor cells, different concentrations of human CCL5 (Peprotech, 300–06-20), or the conditioned medium (CM) samples from stable CRC cell lines. After 48 h of incubation, fibroblasts that successfully migrated to the lower chamber were fixed with 4% paraformaldehyde and stained with hematoxylin. The number of cells was counted in five random visual fields using a light microscope (Olympus, Japan).

### Human cytokine array

Serum-free CM samples from FHC and the human CRC tumor cell lines HCT-8, HCT116, HCT-15, and SW620 were collected after incubation for 24 h and filtered through a 0.22-μm mesh. The CM samples were added to arrays containing antibodies against 1000 unique cytokines (RayBio, GSH-CAA-X00) and processed according to manufacturer’s instructions.

### RNA extraction and quantitative reverse transcription polymerase chain reaction (qRT-PCR)

Total cellular mRNA was extracted using the TRIzol reagent (TaKaRa, 9109). The Prime-Script RT Reagent Kit with gDNA Eraser (TaKaRa, D6110A) was used to reverse-transcribe mRNA into cDNA. Finally, the SYBR Premix Ex Taq (TaKaRa, RR420) and the Applied Biosystems™ 7500 Fast Real-Time PCR System (Thermo Fisher, USA) were used for qRT-PCR. All mRNA levels were normalized based on GAPDH levels, and the 2^−ΔΔCt^ method was used.

### Enzyme-linked immunosorbent assay (ELISA)

CCL5 supernatant levels in the CM samples from FHC and human CRC tumor cells were measured with ELISA using a commercially available kit (CUSABIO, CSB-E17375h), as described by the manufacturer’s instructions. The results were expressed in pg/ml, and the standard curve was based on the measured OD values of the standard.

### siRNA transfection

siRNAs for human CCL5, CCR1, CCR3, CCR4, CCR5, CD44, GPR75, and SLC25A24 were purchased from GenePharma (Suzhou, China). HCT-8, SW620, CCD-18Co, and human primary normal colorectal fibroblasts were transiently transfected with siRNA using the Lipofectamine 3000 Transfection Reagent (Invitrogen, L3000-015) based on the manufacturer’s instructions.

### Construction of stable cell lines

The lentivirus vector LV17 (EF-1a/Luciferase17&Puro) carrying the human CCL5-overexpressing sequence (*CCL5*) and the lentivirus vector LV16 (U6/Firefly&Puro) carrying the indicated CCL5-repressing short hairpin RNA (shRNA) sequence (GGGTTCGGGAGTACATCAA) (*shCCL5*) were obtained from GenePharma (Suzhou, China). Empty LV17 and LV16 vectors served as controls for the overexpression (*Vec*) and repression vectors (*shCtrl*), respectively. Based on the manufacturer’s instructions, stable cell lines were established by transfecting human CRC cell lines with these lentiviral vectors.

### Orthotopic CRC xenograft mouse model

BALB/C-nude mice (male, 3–5 weeks old) were purchased from GemPharmatech Co., Ltd (Guangdong, China). They were housed under specific pathogen-free conditions in the animal facility at the Shenzhen Hospital, Southern Medical University, China. All animal experiments were approved by the ethics committee of Shenzhen Hospital, Southern Medical University, China. First, 5 × 10^6^ HCT116, SW620/*shCtrl*, or SW620/*shCCL5* cells were subcutaneously injected into the backs of nude mice (*n* = 3). After 2 weeks, the IVIS Spectrum In Vivo Imaging System (PerkinElmer, USA) was used to image tumor progression in mice with SW620/*shCtrl* and SW620/*shCCL5* xenografts*.* Further, 15 mg/ml D-Luciferin potassium salts (Promega, P1043) were intraperitoneally administered to the mice (dose, 10 μl/g). The mice were sacrificed, and tumors were surgically removed, fixed in 10% formalin, embedded in paraffin, and cut into 2.5-μm-thick sections for hematoxylin–eosin (H&E) staining.

After the subcutaneous tumors of HCT116 formed, one part of tumor tissues was fixed and stained with H&E, and the remaining tumor tissues were removed and cut into 1-mm^3^ pieces using ophthalmic scissors. Of these pieces, five were chosen and buried inside the cecal serosal layer in nude mice using purse string sutures (*n* = 5). After 8 weeks, the ceca of nude mice were surgically removed and processed as mentioned above.

### Immunoblot/western blot (WB)

Total proteins were isolated from cells using the RIPA lysis buffer (FDbio, FD008), PMSF (FDbio, FD0100), Protease inhibitors (FDbio, FD1001), and protein phosphatase inhibitors (FDbio, FD1002). The concentration was determined using BCA protein assay kits (FDbio, FD2001). The total proteins were separated using 10% sodium dodecyl sulfate–polyacrylamide gel electrophoresis (SDS-PAGE) and transferred to polyvinylidene fluoride (PVDF) membranes. The membranes were blocked with 5% skim milk (FDbio, FD0080) or 5% BSA (FDbio, FD0030) for 1 h at 25 °C and then incubated with primary antibodies at 4 °C overnight. Subsequently, the membranes were incubated with a goat anti-rabbit or anti-mouse secondary antibody (FDbio, FDR007 and FDM007). The proteins were detected using an ECL chemiluminescence solution (FDbio, FD8030) and visualized using a chemiluminescence detection system (Universal Hood II, Bio-Rad). The intensity of each immunoblot band was quantified using the NIH Image J software (National Institutes of Health, USA).

### Treatment with CCR1, CCR5, and Akt inhibitors

Before the recruitment assay, fibroblasts were pre-cultured with a CCR1 inhibitor (*BX471*, 100 nM, MedChemExpress, HY-12080A), CCR5 inhibitor (*Maraviroc*, 100 nM, Selleck, S2003), or Akt inhibitor (*MK-2206*, 2 μM, Selleck, S1078) for 48 h. During the recruitment assay, *BX471*, *Maraviroc*, or *MK-2206* was added to both the upper and lower chambers.

### Transcriptome sequencing

Total mRNA was extracted from fibroblasts before and after treatment with 40 ng/ml CCL5 for 24 h. Sequencing libraries were generated using the NEBNext® UltraTM RNA Library Prep Kit for Illumina® (NEB, USA) following the manufacturer’s recommendations, and index codes were added to attribute the sequences to each sample. The clustering of the index-coded samples was performed on a cBot Cluster Generation System using the TruSeq PE Cluster Kit v3-cBot-HS (Illumina) according to the manufacturer’s instructions.

### Matrigel angiogenesis experiment

First, the high-concentration Matrigel (BD, 354,248) was diluted to half the original concentration. Then, 10 μl of this diluted Matrigel was added to each well of a µ-Slide Angiogenesis Glass Bottom (Ibidi, 81,506) and allowed to polymerize for 30 min at 37 °C. Subsequently, 50 μl medium containing 1 × 10^4^ fibroblasts (treated or not treated with 40 ng/ml CCL5 for 24 h) was incubated in the diluted high-concentration Matrigel. After 2 h, the fibroblasts were fixed with 4% paraformaldehyde and stained with hematoxylin. The number of lumens was obtained across three random visual fields using a light microscope, and capillary tubes were quantified by counting the number of lumens.

### Flow cytometry (Flow-Cyt)

A 4 °C centrifuge was pre-cooled, and PBS containing 0.1% BSA was prepared in advance. Fibroblasts treated with 40 ng/ml CCL5 for 24 h and untreated fibroblasts were collected and washed with PBS. These cells were incubated with the APC-VE-cadherin antibody or APC-rabbit-IgG antibody at 4 °C for 30 min under dark conditions. Then, the cells were washed again with PBS. Finally, the cells were resuspended in 100 μl of PBS, and the cell suspension was transferred to a flow cytometry tube for detection. The fluorescence intensity of fluorescein isothiocyanate was quantified using the Flow-jo software (BD, USA).

### Sirius red staining

Sirius Red staining was performed on paraffin-embedded sections of human CRC tissue and tumor tissue from mouse caecum using the Sirius Red Staining Kit (LEAGENE, DC0041) based on the manufacturer’s protocol. The degree of staining was observed under a polarized light microscope (Olympus, Japan) and scored independently by three pathologists. The percent positivity of Sirius Red staining was scored from 0 to 4, as follows: 0 (< 5%), 1 (5–25%), 2 (26–50%), 3 (51–75%), and 4 (> 75%). The staining intensity was scored on a 4-point scale, as follows: 0 (no staining), 1 (weak staining, light orange and/or green), 2 (moderate staining, medium orange and/or green), and 3 (strong staining, orange and/or green). Subsequently, the Sirius Red staining score was calculated by multiplying the percent positivity score with the staining intensity score. Accordingly, the level of Sirius Red staining was categorized as low (0–4), medium (5–8), or high (9–12).

### Bioinformatics analysis of relapse-free survival and correlation with mRNA expression in CRC patients

The human CRC microarray profile GSE39582 was used to analyze the correlation of COL1 and COL3 expression with relapse-free survival in CRC patients. The chip platform used in this analysis was the Affymetrix Human Genome U133 Plus 2.0 Array. Based on the median values of COL1 and COL3 mRNA expression, patients were divided into low and high expression groups. Each group contained mRNA values for 283 patients. Then, the survival curves of the two groups were obtained using the Kaplan–Meier method. In addition, the human CRC microarray profile GSE39582 was also used to analyze the correlation of CCL5 expression with COL1 and COL3 expression.

### Scanning electron microscopy

Fresh subcutaneous tumor tissues from nude mice (≤ 3 mm^3^) were surgically removed immediately after the mice were sacrificed. The tissues were promptly added to a fixative solution for electron microscopy (Servicebio, G1102) and incubated for 2 h at room temperature before being transferred to 4 °C for storage. Subsequently, the samples were observed and photographed using a scanning electron microscope (Hitachi, Japan).

### Statistical analysis

SPSS software for Mac OS version 25.0 (IBM, USA) was used for statistical analyses. An unpaired two-tailed Student’s *t* test was used to analyze the differences between two groups. The Mann–Whitney U test was conducted to compare the scores of CCL5 staining or Sirius Red staining between CRC tumor tissues and adjacent normal tissues. Pearson’s χ^2^ test and Spearman’s correlation test were applied to analyze the correlation of CCL5 expression or the level of Sirius Red staining with clinicopathological features. The log-rank test was performed to analyze Kaplan–Meier survival curves. The correlation of CCL5 expression with the level of Sirius Red staining in CRC tissues, and the correlation of CCL5 mRNA expression with COL1 or COL3 mRNA expression in the human CRC microarray profile GSE39582 were analyzed using Spearman’s correlation test. All data were expressed as the mean ± standard deviation (SD). *P* < 0.05 was considered statistically significant (ns, no significance; *, *P* < 0.05; **, *P* < 0.01; ***, *P* < 0.001).

## Results

### CRC tumor cells can recruit fibroblasts to tumor buds

**Fig. 1:**
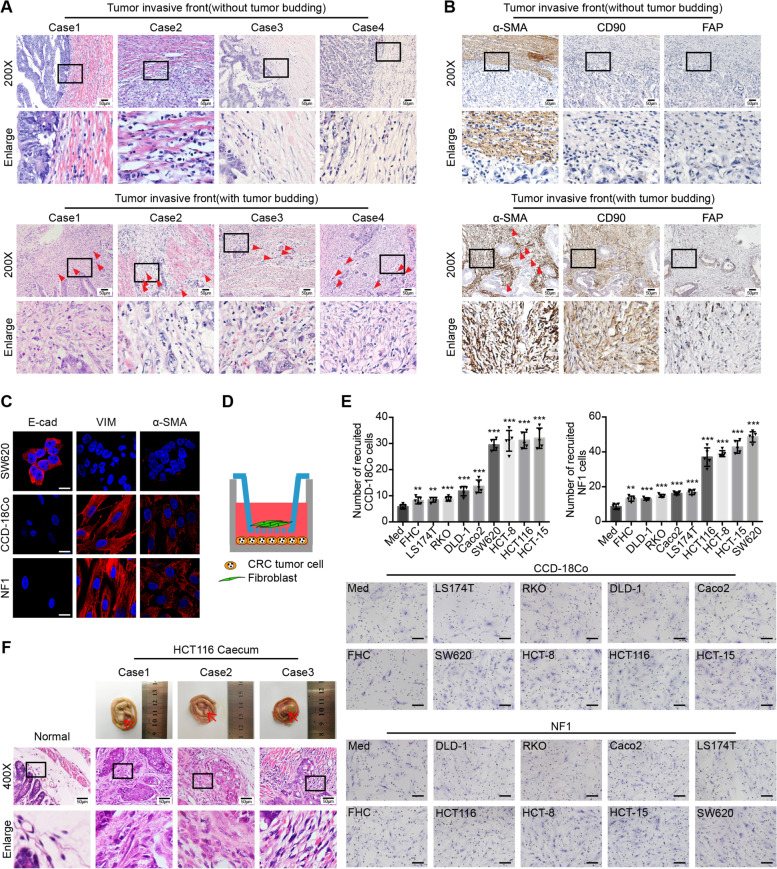
CRC tumor cells can recruit fibroblasts to tumor buds. **a** Representative H&E images of human CRC tissue at the invasive front without (the upper panel) or with (the lower panel) tumor budding. The red triangles indicate the tumor buds. Data, *n* = 195. Scale bar, 50 μm. **b** Representative α-SMA, CD90, and FAP IHC staining images of human CRC tissue at the invasive front without (the upper panel) or with (the lower panel) tumor budding. The red triangles indicate the tumor buds. Data, *n* = 17. Scale bar, 50 μm. **c** Representative E-cad, VIM, and α-SMA IF images of human normal colorectal fibroblast cell line (CCD-18Co) and human primary normal colorectal fibroblast (NF1). Human CRC tumor cell line SW620 is the positive control for E-cad and the negative control for VIM and α-SMA. Scale bar, 20 μm. **d** Model diagram of recruitment assay of CRC tumor cells to fibroblasts. **e** Recruitment assay showing the recruitment ability of medium (Med), human normal colorectal epithelial cell line (FHC) and eight human CRC tumor cell lines (LS174T, RKO, DLD-1, Caco2, SW620, HCT-8, HCT116, HCT-15) to CCD-18Co or human primary normal colorectal fibroblast (NF1). Scale bar, 100 μm. Quantification of cell numbers of recruited fibroblasts is shown in the upper panel. Data, mean ± SD; *n* = 5. **f** Representative morphology images of tumor formation in the mouse caecum (the upper line), and H&E images of normal mouse caecum and tumor tissue in the mouse caecum (the lower two lines). The red arrows indicate the tumors in the mouse caecum. Data, normal, *n* = 5; tumor, *n* = 5. Scale bar, 50 μm. E-cad, E-cadherin; VIM, vimentin. ns, no significance; *, *P* < 0.05; **, *P* < 0.01; ***, *P* < 0.001 by two-tailed Student’s *t* test (**e**)

The evaluation of 195 clinical CRC tissue samples using H&E staining showed that the tumor budding-containing invasive front of CRC tissues had more fibroblasts than other parts of the tumor without tumor budding (Fig. 1a). Next, we verified that the fibroblasts around tumor buds showed a high expression of α-SMA and CD90 but a low expression of FAP (α-SMA^high^ CD90^high^ FAP^low^) (Fig. 1b). These results indicated that tumor budding at the invasive front of CRC tissues was closely related to fibroblast heterogeneity.

Recently, several studies have demonstrated that fibroblasts can be recruited through the effects of cytokines secreted from tumor cells or other cells in the TME [7, 27–29]. While exploring the contribution of CRC cells to fibroblast recruitment, two types of fibroblasts were used: the human normal colorectal fibroblast cell line CCD-18Co and human primary normal colorectal fibroblast, NF1. The human primary normal colorectal fibroblasts were isolated through the enzyme digestion or improved tissue planting method (Additional file 1: Figure S1A). To identify these fibroblasts, the epithelial marker E-cadherin and fibroblast markers vimentin and α-SMA were detected using IF (Fig. 1c and Additional file 1: Figure S1B).

Next, a co-culture recruitment assay was conducted (Fig. 1d). In this assay, human CRC tumor cells were incubated in the lower chamber, while fibroblasts were incubated in the upper chamber. The pore size of the upper co-culture chamber was 8 μm — big enough to allow fibroblasts to pass through. As shown in Fig. 1e, FHC (human normal colorectal epithelial cell line) and LS174T, RKO, DLD-1, and Caco2 (human CRC cell lines) showed a weak ability to recruit fibroblasts. In contrast, HCT-8, HCT116, HCT-15, and SW620 (human CRC cell lines) showed a stronger recruitment ability.

Subsequently, HCT116 cells, which showed a strong ability to recruit fibroblasts in vitro, were used for further in vivo orthotopic CRC xenograft mouse experiments. Using H&E staining to examine tumors in the mouse cecum, we observed that HCT116 could also recruit fibroblasts in vivo (Fig. 1f). Taken together, these data demonstrated that tumor buds of CRC tumors can recruit fibroblasts.

### CRC tumor cells in tumor buds recruit fibroblasts via CCL5

**Fig. 2:**
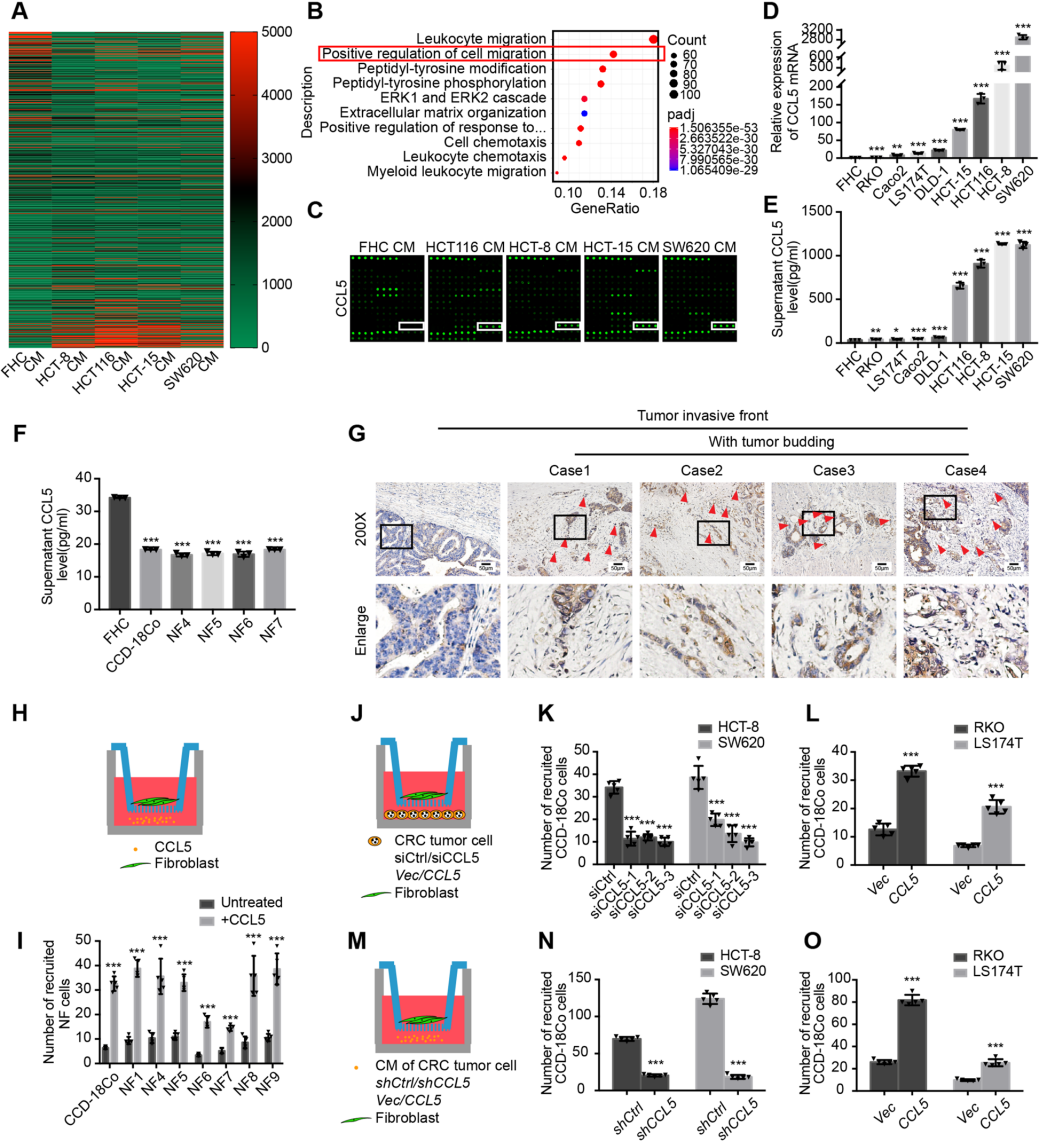
CRC tumor cells in tumor buds recruit fibroblasts via CCL5. **a** Heatmap of the differentially expressed proteins in the cytokine array of the CM samples from FHC (owning a weak recruitment ability) and four human CRC tumor cell lines (HCT-8, HCT116, HCT-15, SW620, owning a strong recruitment ability). The cytokine array detected 1000 cytokines. **b** The differentially expressed proteins in the cytokine were analyzed by GO biological process functional enrichment analysis. **c** Part of the cytokine array including CCL5 showing the expression of CCL5 in the CM samples from FHC and HCT116, HCT-8, HCT-15, SW620. **d** Relative expression of CCL5 mRNA in FHC and eight human CRC tumor cell lines. Data, mean ± SD; *n* = 3. **e** Supernatant CCL5 levels in the CM samples from FHC and eight human CRC tumor cell lines. Data, mean ± SD; *n* = 3. **f** Supernatant CCL5 levels in the CM samples from FHC, CCD-18Co and human primary normal colorectal fibroblasts (NF4, NF5, NF6, NF7). Data, mean ± SD; *n* = 3. **g** Representative CCL5 IHC staining images of human CRC tissue at the invasive front without (the left column) or with (the right four columns) tumor budding. The red triangles indicate the tumor buds. Scale bar, 50 μm. **h** Model diagram of recruitment assay of CCL5 to fibroblasts. **i** Quantification of cell numbers of CCD-18Co and human primary normal colorectal fibroblasts (NF1, NF4, NF5, NF6, NF7, NF8, NF9) recruited by 40 ng/ml CCL5. Data, mean ± SD; *n* = 5. **j** Model diagram of recruitment assay of CCL5 knockdown and overexpressing human CRC tumor cell lines to fibroblasts. **k** Quantification of cell numbers of CCD-18Co recruited by HCT-8/siCtrl and HCT-8/siCCL5, SW620/siCtrl and SW620/siCCL5. Data, mean ± SD; *n* = 5. **l** Quantification of cell numbers of CCD-18Co recruited by RKO/*Vec* and RKO/*CCL5*, LS174T/*Vec* and LS174T/*CCL5*. Data, mean ± SD; *n* = 5. **m** Model diagram of recruitment assay of the CM samples from CCL5 knockdown and overexpressing human CRC tumor cell lines to fibroblasts. **n** Quantification of cell numbers of CCD-18Co recruited by the CM samples from HCT-8/*shCtrl* and HCT-8/*shCCL5*, SW620/*shCtrl* and SW620/*shCCL5*. Data, mean ± SD; *n* = 5. **o** Quantification of cell numbers of CCD-18Co recruited by the CM samples from RKO/*Vec* and RKO/*CCL5*, LS174T/*Vec* and LS174T/*CCL5*. Data, mean ± SD; *n* = 5. ns, no significance; *, *P* < 0.05; **, *P* < 0.01; ***, *P* < 0.001 by two-tailed Student’s *t* test (**d**, **e**, **f**, **i**, **k**, **l**, **n**, **o**)

To further ascertain which key cytokine secreted by CRC tumor cells is responsible for fibroblast recruitment, the CM samples from FHC, which had a weak ability to recruit fibroblasts, and the CM samples from HCT-8, HCT116, HCT-15, and SW620 (human CRC cell lines), which possessed a stronger recruitment ability, were analyzed using a human cytokine array (Fig. 2a). GO functional enrichment analysis indicated that the proteins differentially expressed between these groups were involved in various activities, including “positive regulation of cell migration” (Fig. 2b) and “extracellular matrix” (Additional file 2: Figure S2A). This suggested that cytokines secreted by CRC tumor cells could recruit other cells in the TME and could be involved in the recruitment of fibroblasts, which are the main source of extracellular matrix [1].

Among the differentially expressed proteins, CCL5 showed the most up-regulation in CM samples from human CRC tumor cells (Fig. 2c). The mRNA and secreted protein levels of CCL5 were examined in FHC and eight human CRC tumor cell lines, and the results were entirely consistent with initial findings from the co-culture recruitment assay (Fig. 2d, e). Furthermore, CCD-18Co and human primary normal colorectal fibroblasts were found to secrete lower levels of CCL5 than FHC (Fig. 2f). Accordingly, it appeared that the CCL5-mediated recruitment of fibroblasts occurred through paracrine and not autocrine signaling. Next, the expression of CCL5 was detected in human CRC tissues using IHC, and the results showed that CCL5 was highly expressed in tumor buds at the invasive front (Fig. 2g).

Then, to examine whether CCL5 is the key cytokine for fibroblast recruitment, another recruitment assay was performed (Fig. 2h). Among the different concentrations of CCL5, 40 ng/ml CCL5 showed the strongest ability to recruit fibroblasts (Additional file 2: Figure S2B). At this concentration, CCL5 could recruit CCD-18Co and different human primary normal colorectal fibroblasts (Fig. 2i and Additional file 2: Figure S2C).

Finally, to ascertain whether the CCL5 that recruits fibroblasts was secreted by CRC tumor cells, CCL5 knockdown and overexpressing cell lines were established using siRNA and lentiviral constructs, respectively. The CCL5 mRNA and protein secretion levels in these cell lines were verified (Additional file 2: Figure S2D-G). A co-culture recruitment assay (Fig. 2j) showed that the ability of tumor cells to recruit fibroblasts was significantly weakened after CCL5 knockdown (Fig. 2k and Additional file 2: Figure S2H). In contrast, this ability was enhanced when CCL5 was overexpressed (Fig. 2l and Additional file 2: Figure S2I). In the recruitment assay, the CM samples from stable cells (Fig. 2M and Additional file 2: Figure S2J, K) showed effects similar to those observed with whole cells (Fig. 2N, O and Additional file 2: Figure S2L, M). Together, these results revealed that CCL5 was highly expressed in CRC tumor buds and could recruit fibroblasts from the TME.

### CCL5 recruits fibroblasts through the CCR5 receptor

**Fig. 3:**
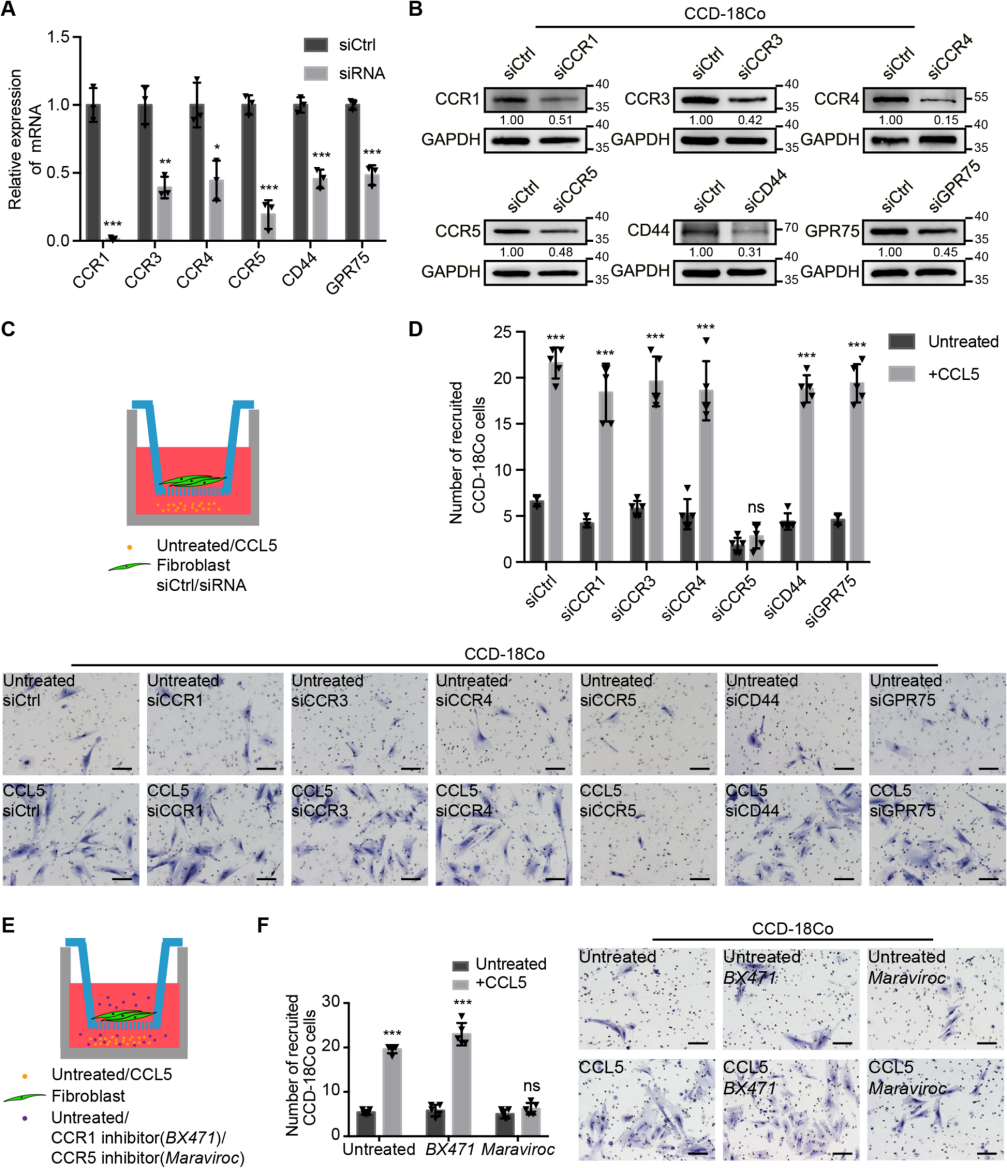
CCL5 recruits fibroblasts through the CCR5 receptor. **a** Relative expression of CCR1, CCR3, CCR4, CCR5, CD44, GPR75 mRNA in CCD-18Co/siCtrl and CCD-18Co/siRNA. Data, mean ± SD; *n* = 3. **b** Immunoblots for CCR1, CCR3, CCR4, CCR5, CD44, GPR75 protein expression in CCD-18Co/siCtrl and CCD-18Co/siRNA. **c** Model diagram of recruitment assay of 40 ng/ml CCL5 to CCR1, CCR3, CCR4, CCR5, CD44, or GPR75 knockdown fibroblasts. **d** Recruitment assay showing the recruitment ability of 40 ng/ml CCL5 to CCD-18Co/siCtrl and CCD-18Co/siRNA. Scale bar, 100 μm. Quantification of cell numbers of recruited CCD-18Co cells is shown in the upper panel. Data, mean ± SD; *n* = 5. **e** Model diagram of recruitment assay of 40 ng/ml CCL5 to fibroblasts without or with CCR1 inhibitor (*BX471*) or CCR5 inhibitor (*Maraviroc*). **f** Recruitment assay showing the recruitment ability of 40 ng/ml CCL5 to CCD-18Co without or with *BX471* or *Maraviroc*. Scale bar, 100 μm. Quantification of cell numbers of recruited CCD-18Co cells is shown in the left panel. Data, mean ± SD; *n* = 5. ns, no significance; *, *P* < 0.05; **, *P* < 0.01; ***, *P* < 0.001 by two-tailed Student’s *t* test (**a**, **d**, **f**)

CCR1, CCR3, CCR4, CCR5, CD44, and GPR75 are known to be possible receptors of CCL5 [18, 19]. Accordingly, to explore whether CCL5 mediates fibroblast recruitment through these receptors, siRNA was used to knock down their expression respectively in fibroblasts, and the interference efficiency was verified (Fig. 3a, b). Next, recruitment assays were performed, as described in Fig. 3c. Surprisingly, only CCR5 downregulation in fibroblasts could significantly attenuate the ability of CCL5 to recruit fibroblasts (Fig. 3d).

Further, the CCR1 inhibitor *BX471* and CCR5 inhibitor *Maraviroc* were used to treat fibroblasts and blocking CCR1 and CCR5, respectively, before and during the co-culture recruitment assay (Fig. 3e). Only *Maraviroc* treatment significantly weakened the ability of CCL5 to recruit fibroblasts (Fig. 3f). The above results strongly indicated that CCL5 was involved in fibroblast recruitment through the CCR5 receptor.

### CCL5-dependent fibroblast recruitment is mediated by SLC25A24 in fibroblasts

**Fig. 4:**
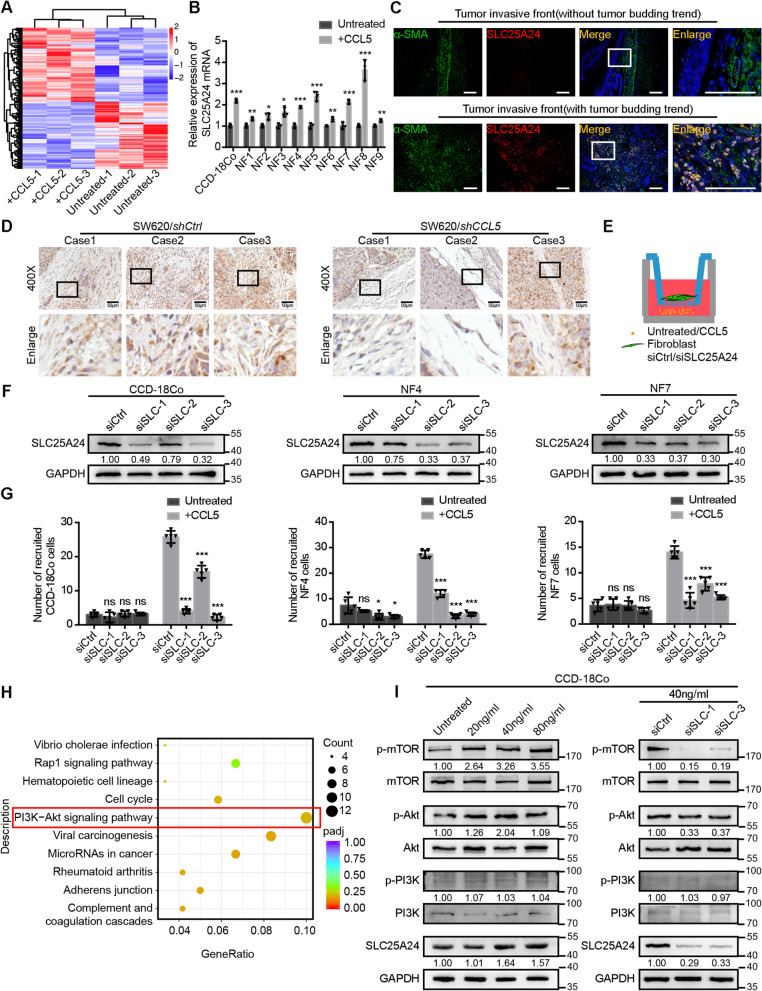
CCL5-dependent fibroblast recruitment is mediated by SLC25A24 in fibroblasts. **a** Heatmap of the differentially expressed genes in the transcriptome sequencing of CCD-18Co before and after 40 ng/ml CCL5 stimulation for 24 h. Data, *n* = 3. **b** Relative expression of SLC25A24 mRNA in CCD-18Co and human primary normal colorectal fibroblasts (NF1, NF2, NF3, NF4, NF5, NF6, NF7, NF8, NF9) before and after 40 ng/ml CCL5 stimulation for 24 h. Data, mean ± SD; *n* = 3. **c** Representative α-SMA and SLC25A24 IF images at the invasive front of human CRC tissue without (the upper panel) or with (the lower panel) tumor budding trend. Data, *n* = 10. Scale bar, 100 μm. **d** Representative SLC25A24 IHC staining images of mouse subcutaneous tumors with the injection of SW620/*shCtrl* or SW620/*shCCL5*. Data, *n* = 3. Scale bar, 50 μm. **e** Model diagram of recruitment assay of 40 ng/ml CCL5 to SLC25A24 knockdown fibroblasts. **f** Immunoblots for SLC25A24 protein expression of CCD-18Co/siCtrl and CCD-18Co/siSLC25A24, NF4/siCtrl and NF4/siSLC25A24, NF7/siCtrl and NF7/siSLC25A24. **g** Quantification of cell numbers of CCD-18Co/siCtrl and CCD-18Co/siSLC25A24, NF4/siCtrl and NF4/siSLC25A24, NF7/siCtrl and NF7/siSLC25A24 recruited by 40 ng/ml CCL5. Data, mean ± SD; *n* = 5. **h** The differentially expressed genes in the transcriptome sequencing were analyzed by KEGG enrichment analysis. **i** Immunoblots for SLC25A24, PI3K, p-PI3K, Akt, p-Akt, mTOR, p-mTOR protein expression in CCD-18Co before and after different concentration of CCL5 stimulation for 24 h (the left panel) and those proteins in CCD-18Co/siCtrl and CCD-18Co/siSLC25A24 after 40 ng/ml CCL5 stimulation for 24 h (the right panel). SLC, SLC25A24; p-PI3K, phosphorylated PI3K; p-Akt, phosphorylated Akt; p-mTOR, phosphorylated mTOR. ns, no significance; *, *P* < 0.05; **, *P* < 0.01; ***, *P* < 0.001 by two-tailed Student’s *t* test (**b**, **g**)

In order to explore the intracellular changes in fibroblasts after CCL5 stimulation, fibroblasts were examined using transcriptome sequencing before and after CCL5 treatment (Fig. 4a). The top 20 differentially expressed genes were selected and verified in CCD-18Co and human primary normal colorectal fibroblasts before and after CCL5 treatment. Among the selected genes, SLC25A24 was found to be consistently up-regulated (Additional file 3: Figure S3A). We subsequently verified that the levels of SLC25A24 mRNA were up-regulated in more types of human primary normal colorectal fibroblasts after CCL5 treatment (Fig. 4b).

SLC25A24, also known as ATP-Mg^2+^/phosphate carrier 1 (APC1), has a regulatory N-terminal domain containing EF-hand Ca^2+^ binding sites, which allow transport in response to cytosolic Ca^2+^ elevations [30–32]. To detect the protein expression and localization of SLC25A24 in human CRC tissues, IF assays were performed. The results revealed that SLC25A24 was highly expressed in the fibroblasts surrounding the tumor buds at the invasive front (Fig. 4c). In vivo, in tumor xenografts of *shCCL5*-transfected CRC cells, reduced SLC25A24 expression was observed in fibroblasts (Fig. 4d). Furthermore, tumor progression was also inhibited (Additional file 3: Figure S3B). Next, we further verified that CCL5 could up-regulate the expression of SLC25A24 protein in vitro (Additional file 3: Figure S3C).

Functionally, to explore whether CCL5-mediated fibroblast recruitment was dependent on SLC25A24, siRNA against SLC25A24 was transfected into fibroblasts, and the recruitment assay was performed (Fig. 4e). A reduction of SLC25A24 expression in fibroblasts significantly attenuated the ability of CCL5 to recruit fibroblasts (Fig. 4f, g and Additional file 3: Figure S3D). Moreover, immunoblot results demonstrated that elevations of SLC25A24 expression in fibroblasts after CCL5 stimulation were dependent on CCR5 (Additional file 3: Figure S3E). This demonstrated CCL5-mediated fibroblast recruitment was dependent on SLC25A24 and CCR5.

To further ascertain which pathways were activated by CCL5 and SLC25A24 in fibroblasts, KEGG enrichment analysis was performed using transcriptome sequencing data. The PI3K-Akt signaling pathway was shown to be the most active after CCL5 stimulation (Fig. 4h). Immunoblot results showed that CCL5 could promote the phosphorylation of Akt and mTOR in fibroblasts (Fig. 4i, left panel). In contrast, when SLC25A24 expression was inhibited, the levels of phosphorylated Akt and mTOR decreased in fibroblasts after CCL5 stimulation (Fig. 4i, right panel). Furthermore, inhibiting Akt phosphorylation in fibroblasts reduced the fibroblast recruitment ability of CCL5 (Additional file 3: Figure S3F). However, this inhibition had no effect on the expression of SLC25A24 (Additional file 3: Figure S3G). These findings suggested that CCL5 recruited fibroblasts through the SLC25A24-pAkt-pmTOR axis within fibroblasts.

### CCL5 contributes to the increase in α-SMA^high^ CD90^high^ FAP^low^ fibroblasts and thereby promotes tumor angiogenesis

**Fig. 5:**
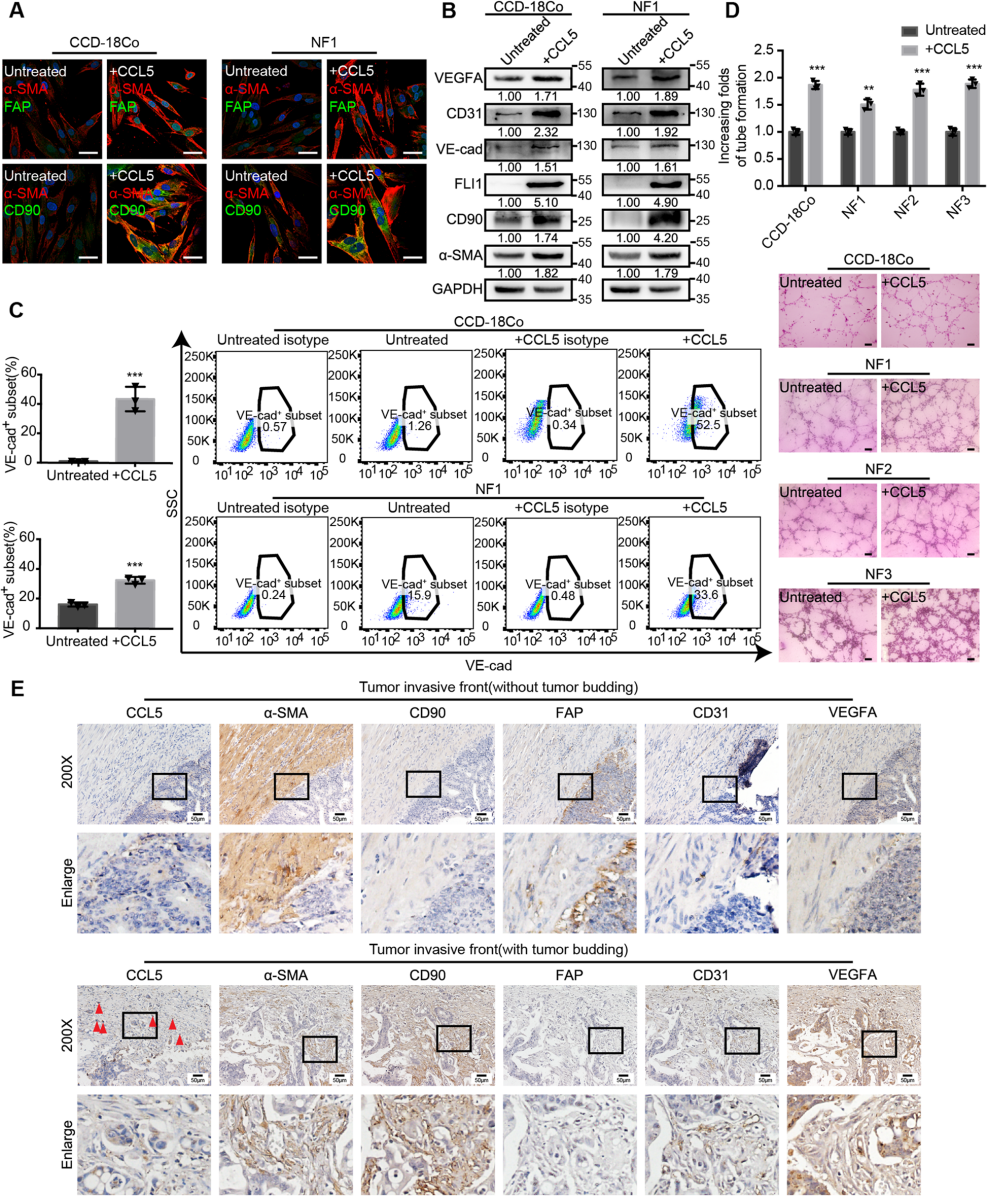
CCL5 contributes to the increase in α-SMA^high^ CD90^high^ FAP^low^ fibroblasts and thereby promotes tumor angiogenesis. **a** Representative α-SMA, FAP and CD90 IF images of CCD-18Co and human primary normal colorectal fibroblast (NF1) before and after 40 ng/ml CCL5 stimulation for 24 h. Scale bar, 40 μm. **b** Immunoblots for α-SMA, CD90, FLI1, VE-cad, CD31 and VEGFA protein expression in CCD-18Co and human primary normal colorectal fibroblast (NF1) before and after 40 ng/ml CCL5 stimulation for 24 h. **c** Flow cytometry showing the proportion of VE-cad^+^ subset in CCD-18Co and human primary normal colorectal fibroblast (NF1) before and after 40 ng/ml CCL5 stimulation for 24 h. Quantification is shown in the left panel. Data, mean ± SD; *n* = 3. **d** Matrigel angiogenesis experiments showing the angiogenesis ability of CCD-18Co and human primary normal colorectal fibroblasts (NF1, NF2, NF3) before and after 40 ng/ml CCL5 stimulation. Scale bar, 100 μm. Quantification of increasing folds of tube formation is shown in the upper panel. Data, mean ± SD; *n* = 3. **e** Representative CCL5, α-SMA, CD90, FAP, CD31 and VEGFA IHC staining images of human CRC tissue at the invasive front without (the upper panel) or with (the lower panel) tumor budding. The red triangles indicate the tumor buds. Data, *n* = 10. Scale bar, 50 μm. VE-cad, VE-cadherin. ns, no significance; *, *P* < 0.05; **, *P* < 0.01; ***, *P* < 0.001 by two-tailed Student’s *t* test (**c**, **d**)

Our initial findings (Fig. 1b) proved that fibroblasts around tumor buds were α-SMA^high^, CD90^high^, and FAP^low^. To examine whether CCL5 is the main contributor to the increase in α-SMA^high^ CD90^high^ FAP^low^ fibroblasts, an IF assay was performed. This assay showed that the expression of α-SMA and CD90 was elevated in fibroblasts treated with CCL5 (Fig. 5a). VEGFA is necessary for the proliferation, survival, migration, and invasion of vascular endothelial cells into surrounding tissue and the subsequent generation of lumen-containing structures [33]. It has been reported that fibroblasts are one of the main sources of VEGFA [2]. Furthermore, studies have shown that fibroblasts can transdifferentiate into vascular endothelial cells and facilitate tumor progression [34–36]. We examined the role of CCL5 in fibroblast-mediated angiogenesis. Immunoblots showed that the levels of the vascular endothelial markers FLI1, VE-cadherin, CD31, and VEGFA as well as those of α-SMA and CD90 were elevated in fibroblasts treated with CCL5 (Fig. 5b). Flow cytometry also demonstrated that the proportion of the VE-cadherin^+^ subset of fibroblasts increased after CCL5 stimulation (Fig. 5c and Additional file 4: Figure S4A). Functionally, the proangiogenic ability of fibroblasts was also found to be enhanced after CCL5 stimulation (Fig. 5d). Serial sections from 10 clinical CRC samples were stained for CCL5, α-SMA, CD90, FAP, CD31, and VEGFA using IHC. At the invasive front, CCL5 was highly expressed in tumor buds, which were surrounded by a high number of α-SMA^high^ CD90^high^ FAP^low^ fibroblasts and blood vessels (Fig. 5e). Immunoblots and Matrigel angiogenesis experiments showed that when fibroblasts were transfected with SLC25A24 siRNA, there was no attenuation of the CCL5-mediated increase in angiogenesis (Additional file 4: Figure S4B, C). Based on these findings, we speculated that tumor bud-derived CCL5 increased the number of α-SMA^high^ CD90^high^ FAP^low^ fibroblasts and promoted tumor angiogenesis via the increase in VEGFA and the transdifferentiation of fibroblasts into vascular endothelial cells.

### CCL5 promotes collagen synthesis via fibroblasts, contributing to tumor progression

**Fig. 6:**
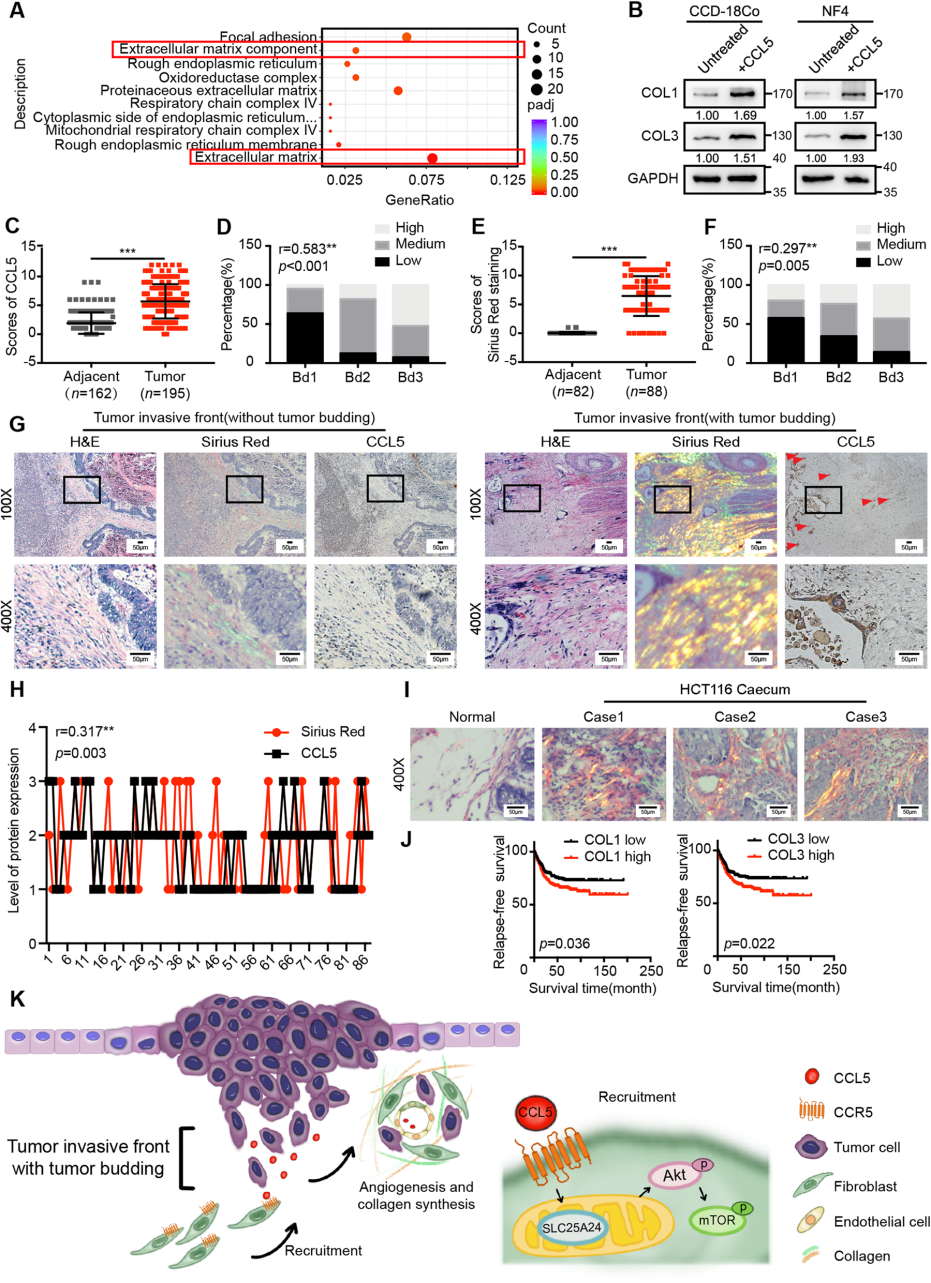
CCL5 promotes collagen synthesis via fibroblasts, contributing to tumor progression. **a** The differentially expressed genes in the transcriptome sequencing were analyzed by GO functional enrichment analysis. **b** Immunoblots for COL1 and COL3 protein expression in CCD-18Co and human primary normal colorectal fibroblast (NF4) before and after 40 ng/ml CCL5 stimulation for 24 h. **c** IHC scores of CCL5 in human adjacent normal tissues and CRC tumor tissues. Data, adjacent, *n* = 162; tumor, *n* = 195. (**d**) Spearman’s correlation analysis of low and high expression of CCL5 in human CRC patients with the different tumor budding categories. Spearman *r* = 0.583. Data, *n* = 195. **e** Scores of Sirius Red staining in human adjacent normal tissues and CRC tumor tissues. Data, adjacent, *n* = 82; tumor, *n* = 88. **f** Spearman’s correlation analysis of low and high level of Sirius Red staining in human CRC patients with the different tumor budding categories. Spearman r = 0.297. Data, *n* = 88. **g** Representative CCL5 IHC staining and Sirius Red staining images of human CRC tissue at the invasive front without (the left panel) or with (the right panel) tumor budding. The red triangles indicate the tumor buds. Scale bar, 50 μm. **h** Spearman’s correlation analysis between the level of CCL5 expression and Sirius Red staining in human CRC tissues. Spearman r = 0.317. Data, *n* = 88. **i** Representative Sirius Red staining images of normal mouse caecum and tumor tissue in the mouse caecum. Data, normal, *n* = 5; tumor, *n* = 5. Scale bar, 50 μm. **j** Kaplan–Meier survival analysis of CRC patients with low and high expression of COL1 mRNA or COL3 mRNA in the human CRC microarray profile GSE39582. Data, *n* = 566. **k** Schematic diagram of the contribution of tumor bud-derived CCL5 to the TME at the invasive front. ns, no significance; *, *P* < 0.05; **, *P* < 0.01; ***, *P* < 0.001 by Mann–Whitney U test (**c**, **e**), Spearman’s correlation test (**d**, **f**, **h**), log-rank test (**j**)

GO functional enrichment analysis performed using transcriptome sequencing data indicated that CCL5 was also involved in extracellular matrix-related functions (Fig. 6a). It is well-established that collagen type I (COL1) and collagen type III (COL3) are the primary components of the extracellular matrix and are mainly synthesized and secreted by fibroblasts [2]. To clarify the role of CCL5 in collagen synthesis, immunoblots were performed. The findings demonstrated that CCL5 could increase the protein expression of COL1 and COL3 in fibroblasts in vitro (Fig. 6b).

To further investigate the role of CCL5 in CRC, the expression of the CCL5 protein was examined in 195 paraffin-embedded human CRC tissue samples and 162 human adjacent normal colorectal tissue samples. CCL5 expression was significantly higher in CRC tissues than in normal tissues (Fig. 6c and Additional file 5: Figure S5A). The correlation between CCL5 expression and clinical features was analyzed using Pearson’s χ^2^ test (Table 1). Further Spearman’s correlation tests showed that high levels of CCL5 expression were positively associated with a high risk of increased tumor buds (*r* = 0.583, *P* < 0.001, Fig. 6d), deep tumor invasion (*r* = 0.244, *P* = 0.001), lymph node metastasis (*r* = 0.237, *P* = 0.001), the presence of peri-intestinal cancer nodule deposition (*r* = 0.198, *P* = 0.005), and advanced TNM stages (*r* = 0.256, *P* < 0.001) (Additional file 5: Figure S5B-E). Moreover, Sirius Red staining for COL1 (reddish) and COL3 (greenish) was performed to detect the collagen distribution in 88 of the 195 human CRC tissue samples. The expression of COL1 and COL3 was elevated in CRC tissue (Fig. 6e; Additional file 5: Figure S5F). The correlation between Sirius Red staining and the clinical features of CRC was also analyzed using Pearson’s χ^2^ test (Table 2). Further Spearman’s correlation tests showed that a high collagen distribution around CRC tumor cells was positively related to increased tumor buds (*r* = 0.297, *P* = 0.005, Fig. 6f), deep tumor invasion (*r* = 0.431, *P* = 0.001), lymph node metastasis (*r* = 0.351, *P* = 0.001), the presence of peri-intestinal cancer nodule deposition (*r* = 0.288, *P* = 0.007), and advanced TNM stages (*r* = 0.442, *P* = 0.001) (Additional file 5: Figure S5G-J). Intriguingly, the high expression of CCL5 in tumor buds at the invasive front was often accompanied by increased collagen synthesis (Fig. 6g). Spearman’s correlation analyses revealed a positive correlation between the levels of CCL5 expression in tumor cells and the amounts of collagen in the surrounding area from the same CRC tissue sample (*n* = 88; *r* = 0.317, *P* = 0.003, Fig. 6h).

**Table 1:** The relationship between CCL5 expression and CRC clinicopathological features

	No. of cases	Low	Medium	High	*x*^*2*^ value	***P***-value
**Frequency**	195 (100%)	65 (33.3%)	92 (47.2%)	38 (19.5%)		
**Age**						
< 60 years	96 (49.2%)	33 (34.4%)	40 (41.7%)	23 (24.0%)	**3.219**	**0.200**
> = 60 years	99 (50.8%)	32 (32.3%)	52 (52.5%)	15 (15.2%)		
**Gender**						
Male	119 (61.0%)	43 (36.1%)	50 (42.0%)	26 (21.8%)	**3.317**	**0.190**
Female	76 (39.0%)	22 (28.9%)	42 (55.3%)	12 (15.8%)		
**Position**						
Colon	128 (65.6%)	41 (32.0%)	61 (47.7%)	26 (20.3%)	**2.471**	**0.650**
Rectum	58 (29.7%)	20 (34.5)	26 (44.8%)	12 (20.7%)		
Colorectum	9 (4.6%)	4 (44.4%)	5 (55.6%)	0 (0.0%)		
**Tumor size(maximum diameter)**						
< 5 cm	99 (51.0%)	30 (30.3%)	52 (52.5%)	17 (17.2%)	**2.155**	**0.340**
> = 5 cm	95 (49.0%)	34 (35.8%)	40 (42.1%)	21 (22.1%)		
**Histology**						
Poor	22 (11.3%)	6 (27.3%)	13 (59.1%)	3 (13.6%)	**1.505**	**0.826**
Moderate	155 (79.5%)	53 (34.2%)	71 (45.8%)	31 (20.0%)		
Well	18 (9.2%)	6 (33.3%)	8 (44.4%)	4 (22.2%)		
**T stage**						
Tis	2 (1.0%)	2 (100.0%)	0 (0.0%)	0 (0.0%)	**22.460**	**0.004**
T1	4 (2.1%)	3 (75.0%)	1 (25.0%)	0 (0.0%)		
T2	21 (10.8%)	6 (28.6%)	11 (52.4%)	4 (19.0%)		
T3	123 (63.1%)	47 (38.2%)	59 (48.0%)	17 (13.8%)		
T4	45 (23.1%)	7 (15.6%)	21 (46.7%)	17 (37.8%)		
**Tumor budding**						
Bd1	83 (42.6%)	53 (63.9%)	26 (31.3%)	4 (4.8%)	**84.614**	** < 0.001**
Bd2	72 (36.9%)	9 (12.5%)	50 (69.4%)	13 (18.1%)		
Bd3	40 (20.5%)	3 (7.5%)	16 (40.0%)	21 (52.5%)		
**Lymphatic metastasis**						
Negative	107 (54.9%)	46 (43.0%)	46 (43.0%)	15 (14.0%)	**11.154**	**0.004**
Positive	88 (45.1%)	19 (21.6%)	46 (52.3%)	23 (26.1%)		
**Tumor deposits**						
Absent	171 (87.7%)	61 (35.7%)	82 (48.0%)	28 (16.4%)	**9.366**	**0.009**
Present	24 (12.3%)	4 (16.7%)	10 (41.7%)	10 (41.7%)		
**Mucinous component**						
Absent	162 (83.1%)	53 (32.7%)	77 (47.5%)	32 (19.8%)	**0.169**	**0.919**
Present	33 (16.9%)	12 (36.4%)	15 (45.5%)	6 (18.2%)		
**Distant metastasis**						
Negative	182 (93.3%)	63 (34.6%)	83 (45.6%)	36 (19.8%)	**2.902**	**0.234**
Positive	13 (6.7%)	2 (15.4%)	9 (69.2%)	2 (15.4%)		
**Microstatellite instability**						
MSS	161 (82.6%)	53 (32.9%)	74 (46.0%)	34 (21.1%)	**4.754**	**0.314**
MSI-L	11 (5.6%)	6 (54.5%)	5 (45.5%)	0 (0.0%)		
MSI-H	23 (11.8%)	6 (26.1%)	13 (56.5%)	4 (17.4%)		
**Neoadjuvant chemotherapy**						
Negative	186 (95.4%)	61 (32.8%)	90 (48.4%)	35 (18.8%)	**2.523**	**0.283**
Positive	9 (4.6%)	4 (44.4%)	2 (22.2%)	3 (33.3%)		
**TNM stage**						
0	2 (1.0%)	2(100.0%)	0 (0.0%)	0 (0.0%)	**41.890**	** < 0.001**
I	18 (9.2%)	8 (44.4%)	7 (38.9%)	3 (16.7%)		
IIA	12 (6.2%)	32 (45.7%)	30 (42.9%)	8 (11.4%)		
IIB	70 (35.9%)	2 (22.2%)	4 (44.4%)	3 (33.3%)		
IIIA	4 (2.1%)	1 (25.0%)	2 (50.0%)	1 (25.0%)		
IIIB	56 (28.7%)	15 (26.8%)	34 (60.7%)	7 (12.5%)		
IIIC	24 (12.3%)	3 (12.5%)	7 (29.2%)	14 (58.3%)		
IV	12 (6.2%)	2 (16.7%)	8 (66.7%)	2 (16.7%)

**Table 2:** The relationship between Sirius Red staining and CRC clinicopathological features

	No. of cases	Low	Medium	High	*x*^*2*^ value	***P***-value
**Frequency**	88 (100%)	38 (43.2%)	28 (31.8%)	22 (25%)		
**Age**						
< 60 years	40 (45.5%)	16 (40.0%)	12 (30.0%)	12 (30.0%)	**0.981**	**0.612**
> = 60 years	48 (54.5%)	22 (45.8%)	16 (33.3%)	10 (20.8%)		
**Gender**						
Male	51 (58.0%)	22 (43.1%)	17 (33.3%)	12 (23.5%)	**0.193**	**0.908**
Female	37 (42.0%)	22 (43.2%)	42 (29.7%)	12 (27.0%)		
**Position**						
Colon	57 (64.8%)	21 (36.8%)	20 (35.1%)	16 (28.1%)	**4.218**	**0.377**
Rectum	27 (30.7%)	15 (55.6%)	6 (22.2%)	6 (22.2%)		
Colorectum	4 (4.5%)	2 (50.0%)	2 (50.0%)	0 (0.0%)		
**Tumor size(maximum diameter)**						
< 5 cm	46 (52.3%)	17 (37.0%)	18 (39.1%)	11 (23.9%)	**2.530**	**0.282**
> = 5 cm	42 (47.7%)	21 (50.0%)	10 (23.8%)	11 (26.2%)		
**Histology**						
Poor	13 (14.8%)	6 (46.2%)	2 (15.4%)	5 (38.5%)	**5.948**	**0.203**
Moderate	65 (73.9%)	25 (38.5%)	24 (36.9%)	16 (24.6%)		
Well	10 (11.4%)	7 (70.0%)	2 (20.0%)	1 (10.0%)		
**T stage**						
Tis	1 (1.1%)	1 (100.0%)	0 (0.0%)	0 (0.0%)	**19.325**	**0.013**
T1	3 (3.4%)	3 (100.0%)	0 (0.0%)	0 (0.0%)		
T2	12 (13.6%)	10 (83.3%)	2 (16.7%)	0 (0.0%)		
T3	57 (64.8%)	21 (36.8%)	21 (36.8%)	15 (26.3%)		
T4	15 (17.0%)	3 (20.0%)	5 (33.3%)	7 (46.7%)		
**Tumor budding**						
Bd1	45 (51.1%)	26 (57.8%)	10 (22.2%)	9 (20.0%)	**10.351**	**0.035**
Bd2	29 (33.0%)	10 (34.5%)	12 (41.4%)	7 (24.1%)		
Bd3	14 (15.9%)	2 (14.3%)	6 (42.9%)	6 (42.9%)		
**Lymphatic metastasis**						
Negative	48 (54.5%)	28 (58.3%)	13 (27.1%)	7 (14.6%)	**10.941**	**0.004**
Positive	40 (45.5%)	10 (25.0%)	15 (37.5%)	15 (37.5%)		
**Tumor deposits**						
Absent	75 (85.2%)	36 (48.0%)	24 (32.0%)	15 (20.0%)	**7.812**	**0.020**
Present	13 (14.8%)	2 (15.4%)	4 (30.8%)	7 (53.8%)		
**Mucinous component**						
Absent	17 (19.3%)	7 (41.2%)	5 (29.4%)	5 (29.4%)	**0.222**	**0.895**
Present	71 (80.7%)	31 (43.7%)	23 (32.4%)	17 (23.9%)		
**Distant metastasis**						
Negative	85 (96.6%)	37 (43.5%)	27 (31.8%)	21 (24.7%)	**0.158**	**0.924**
Positive	3 (3.4%)	1 (33.3%)	1 (33.3%)	1 (33.3%)		
**Microstatellite instability**						
MSS	71 (80.7%)	28 (39.4%)	24 (33.8%)	19 (26.8%)	**4.258**	**0.372**
MSI-L	7 (8.0%)	3 (42.9%)	3 (42.9%)	1 (14.3%)		
MSI-H	10 (11.4%)	7 (70.0%)	1 (10.0%)	2 (20.0%)		
**Neoadjuvant chemotherapy**						
Negative	87 (98.9%)	38 (32.8%)	28 (48.4%)	21 (18.8%)	**3.034**	**0.219**
Positive	1 (1.1%)	0 (44.4%)	0 (22.2%)	1 (33.3%)		
**TNM stage**						
0	2 (2.3%)	2(100.0%)	0 (0.0%)	0 (0.0%)	**20.396**	**0.009**
I	10 (11.4%)	9 (90.0%)	1 (10.0%)	0 (0.0%)		
II	31 (35.2%)	16 (51.6%)	10 (32.3%)	5 (16.1%)		
III	42 (47.7%)	10 (23.8%)	16 (38.1%)	16 (38.1%)		
IV	3 (3.4%)	1 (33.3%)	1 (33.3%)	1 (33.3%)		

Subsequently, HCT116 cells, which secrete large amounts of CCL5, were used to establish orthotopic CRC xenograft mouse models for in vivo analysis. We found that collagen synthesis was increased around tumor cells at the invasive front (Fig. 6i). Furthermore, the human CRC microarray profile GSE39582 was used to analyze the relapse-free survival of patients with low and high expression of COL1 or COL3. A higher expression of COL1 and COL3 was closely related to a poor prognosis in CRC patients (Fig. 6j). Spearman’s correlation analyses using GSE39582 also showed that CCL5 mRNA expression was correlated with COL1 mRNA (*r* = 0.211, *P* < 0.001) and COL3 mRNA expression (*r* = 0.228, *P* < 0.001) (Additional file 5: Figure S5K, L). Moreover, immunoblots showed that the transfection of SLC25A24 siRNA in fibroblasts did not attenuate the CCL5-mediated increase in collagen synthesis (Additional file 5: Figure S5M). Additionally, collagen fibers in the tumor xenografts of *shCtrl*-transfected CRC cells were oriented in the same direction, forming thicker collagen fiber bundles. In contrast, the collagen fibers in the tumor xenografts of *shCCL5*-transfected CRC cells were disorganized (Additional file 5: Figure S5N). These findings demonstrated that *shCCL5* substantially attenuated collagen linearization in vivo, which was suggestive of attenuated stiffness and a subsequent reduction in tumor progression [37, 38]. Taken together, these results suggested that tumor buds secreted CCL5, which then promoted collagen synthesis via fibroblasts, thus contributing to tumor progression.

## Discussion

The TME plays multiple roles in tumorigenesis because it harbors cancer cells that interact with surrounding cells and promote cancer progression [39, 40]. Inside the TME, fibroblasts exert strong tumor-modulating effects, which are closely related to disease recurrence and a poor prognosis [41–44]. Although fibroblasts play important roles in CRC development, specific markers for the highly heterogenous and complicated fibroblast population are currently unavailable. The present study revealed that CRC tumor buds secreted high levels of CCL5, which recruited fibroblasts through CCR5-SLC25A24 signaling and led to the development of a characteristic fibroblast cluster around tumor buds at the invasive front. This further facilitated tumor angiogenesis and collagen synthesis, promoting malignant progression (Fig. 6k).

Tumor buds consist of the most aggressive subgroups of tumor cells, which play a leading role in tumor invasion [12, 13]. However, unique markers for tumor budding are unavailable, and H&E staining is not a reliable method for counting tumor buds when tumor cells are difficult to distinguish from reactive mesenchymal cells. In this study, CCL5 was found to be a marker for tumor budding. Moreover, our findings provide concrete new evidence for the tumor-promoting role of tumor budding. Consequently, the findings indicate that the accuracy of the pathological diagnosis of tumor budding in CRC can be increased by examining CCL5 expression. Studies have shown that the high expression of CCL5 in CRC tumor cells can promote their proliferation [45]. The increased secretion of CCL5 from CRC cells can also promote the apoptosis of CD8^+^ T cells via regulatory T cells, thereby promoting tumor progression via immunosuppression [22]. Our study provides more comprehensive insights into the role of CCL5 in CRC progression and elucidates the comprehensive tumor–microenvironment interaction network in CRC. These findings show that therapies targeting CCL5 may play a significant role in blocking CRC progression.

In previous studies, circulating tumor cells have been detected along with fibroblasts, and this finding is highly correlated with tumor metastasis [46–48]. These results suggest that fibroblasts are also critical factors in tumor progression. Thus, the heterogeneity of fibroblasts is also worthy of attention. We found that a subgroup of fibroblasts was stimulated by CCL5. These fibroblasts were α-SMA^high^, CD90^high^, and FAP^low^, and were mainly located around tumor buds. These cells contributed to multiple tumor-promoting processes, including tumor angiogenesis and collagen synthesis. Thus, our findings reveal new mechanisms underlying the effect of tumor budding on tumor progression. Besides, our study found that tumor budding promoted fibroblast recruitment through the CCR5 receptor during invasion, and this was a vital step for further angiogenesis and collagen synthesis. Another study has also demonstrated that tumor immune cells can be targeted effectively during CRC metastasis through clinical anti-CCR5 therapy [49]. Consequently, CCR5 inhibitors can not only target immune cells but also fibroblasts at the invasive front of CRC, indicating their value in CRC treatment. The CCR5 inhibitor *Maraviroc*, is commonly used in the clinical treatment of HIV [50–52], and it could also have benefits for CRC treatment. Nevertheless, further research remains warranted.

The SLC25 carrier family includes 53 members, most of which transport solutes across the inner mitochondrial membrane during various distinct metabolic processes [53]. SLC25A24 belongs to a subgroup of short calcium-binding mitochondrial carriers (SCaMCs) and has four paralogs in mammals: SCaMC-3/SLC25A23, SCaMC-1/SLC25A24, SCaMC-2/SLC25A25, and SCaMC-3-like/SLC25A41 [54–56]. These carriers consist of a C-terminal domain containing six transmembrane helices homologous to mitochondrial carrier proteins, and an N-terminal domain with Ca^2+^-binding EF hands that confer Ca^2+^ sensitivity to the carrier. As a mitochondrial inner membrane protein, SLC25A24 is involved in the uptake and accumulation of adenine nucleotides [57]. In addition, SLC25A24 is also reported to have anti-oxidative effects [58]. In this study, we found that SLC25A24 was highly expressed in fibroblasts surrounding the tumor buds at the invasive front. The specific regulatory interactions between CCR5 and SLC25A24, and the effect of increased SLC25A24 expression on mitochondrial function still require further researches. Further, the effect of these SLC25A24^high^ fibroblasts on tumor cells is still unclear and remains to be understood.

## Conclusions

In conclusion, the present study suggests that at the invasive front of CRC, tumor bud-derived CCL5 can recruit fibroblasts via CCR5-SLC25A24 signaling. Accordingly, tumor bud-derived CCL5 can further promote angiogenesis and collagen synthesis through fibroblasts, and eventually, create a tumor-promoting microenvironment. Therefore, our study provides evidences indicating that CCL5 may serve as a potential diagnostic marker and therapeutic target for tumor budding in CRC.

## Supplementary Information

**Additional file 1: Figure S1.** Extraction and verification of human primary normal colorectal fibroblasts.

**Additional file 2: Figure S2.** CRC tumor cells in tumor buds recruit fibroblasts via CCL5.

**Additional file 3: Figure S3. **CCL5-dependent fibroblast recruitment is mediated by SLC25A24 in fibroblasts.

**Additional file 4: Figure S4. **CCL5 contributes to the increase in α-SMA^high^ CD90^high^ FAP^low^ fibroblasts and thereby promotes tumor angiogenesis.

**Additional file 5: Figure S5.** CCL5 promotes collagen synthesis via fibroblasts, contributing to tumor progression.

**Additional file 6: Table S1.** List of primary antibodies used in the study.

**Additional file 7: Table S2.** List of RNA interference oligo sequences.

**Additional file 8: Table S3.** List of primer sequences.

## Data Availability

All data presented or analyzed in this study are included either in this article or in the additional files.

## Abbreviations

CRC: Colorectal cancer
CCL5: C–C chemokine ligand 5
SLC25A24: Solute carrier family 25 member 24
TME: Tumor microenvironment
CAFs: Cancer-associated fibroblasts
RANTES: Regulated upon Activation, Normal T-cell Expressed, and Secreted
siRNA: Small interfering RNA
shRNA: Short hairpin RNA
ATCC: American Type Culture Collection
FBS: Fetal bovine serum
EMEM: Eagle’s Minimum Essential Medium
ITBCC: International Tumor Budding Consensus Conference
IHC: Immunohistochemistry
IF: Immunofluorescence
ELISA: Enzyme-linked immunosorbent assay
qRT-PCR: Quantitative reverse transcription polymerase chain reaction
CM: Conditioned medium
SDS-PAGE: Sodium dodecyl sulfate–polyacrylamide gel electrophoresis
PVDF: Polyvinylidene fluoride
H&E: Hematoxylin–eosin
Flow-Cyt: Flow cytometry
COL1: Collagen type I
COL3: Collagen type III
APC1: ATP-Mg^2^^+^/phosphate carrier 1
SCaMCs: Short calcium-binding mitochondrial carriers
