# Supplementary figures and images for: Tumor bud-derived CCL5 recruits fibroblasts and promotes colorectal cancer progression via CCR5-SLC25A24 signaling
Source: J Exp Clin Cancer Res. 2022 Mar 3;41:81. doi: 10.1186/s13046-022-02300-w (PMC8892738; doi:10.1186/s13046-022-02300-w)

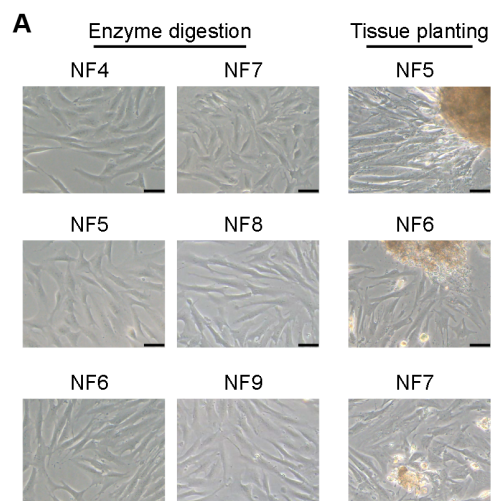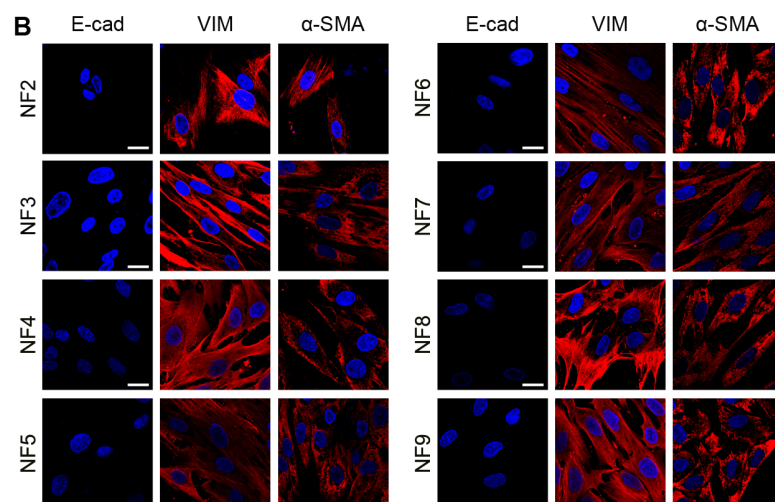

Supplement: Supplementary file 1 — Additional file 1: Figure S1. Extraction and verification of human primary normal colorectal fibroblasts. [file 13046_2022_2300_MOESM1_ESM.pdf]

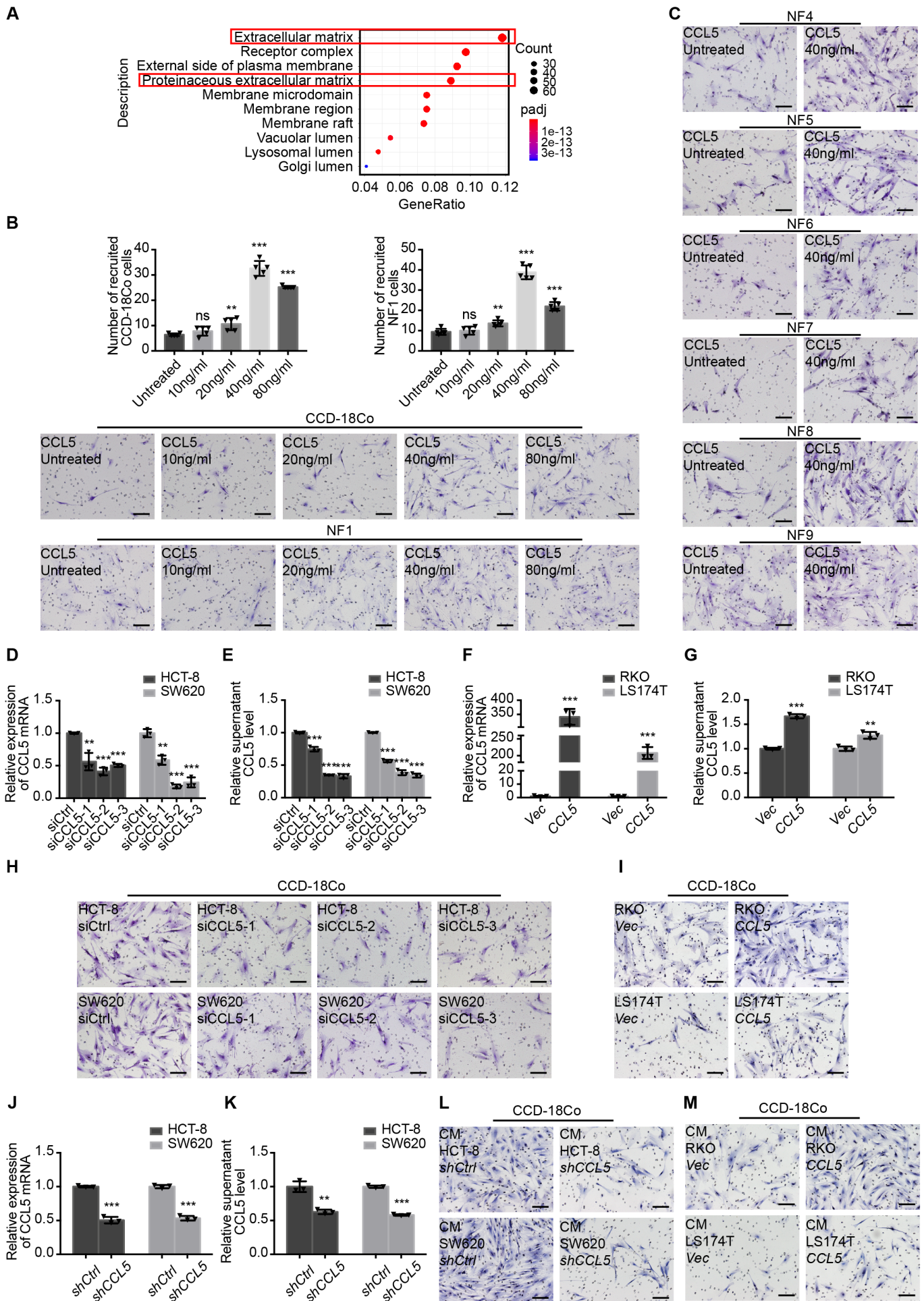

Supplement: Supplementary file 2 — Additional file 2: Figure S2. CRC tumor cells in tumor buds recruit fibroblasts via CCL5. [file 13046_2022_2300_MOESM2_ESM.pdf]

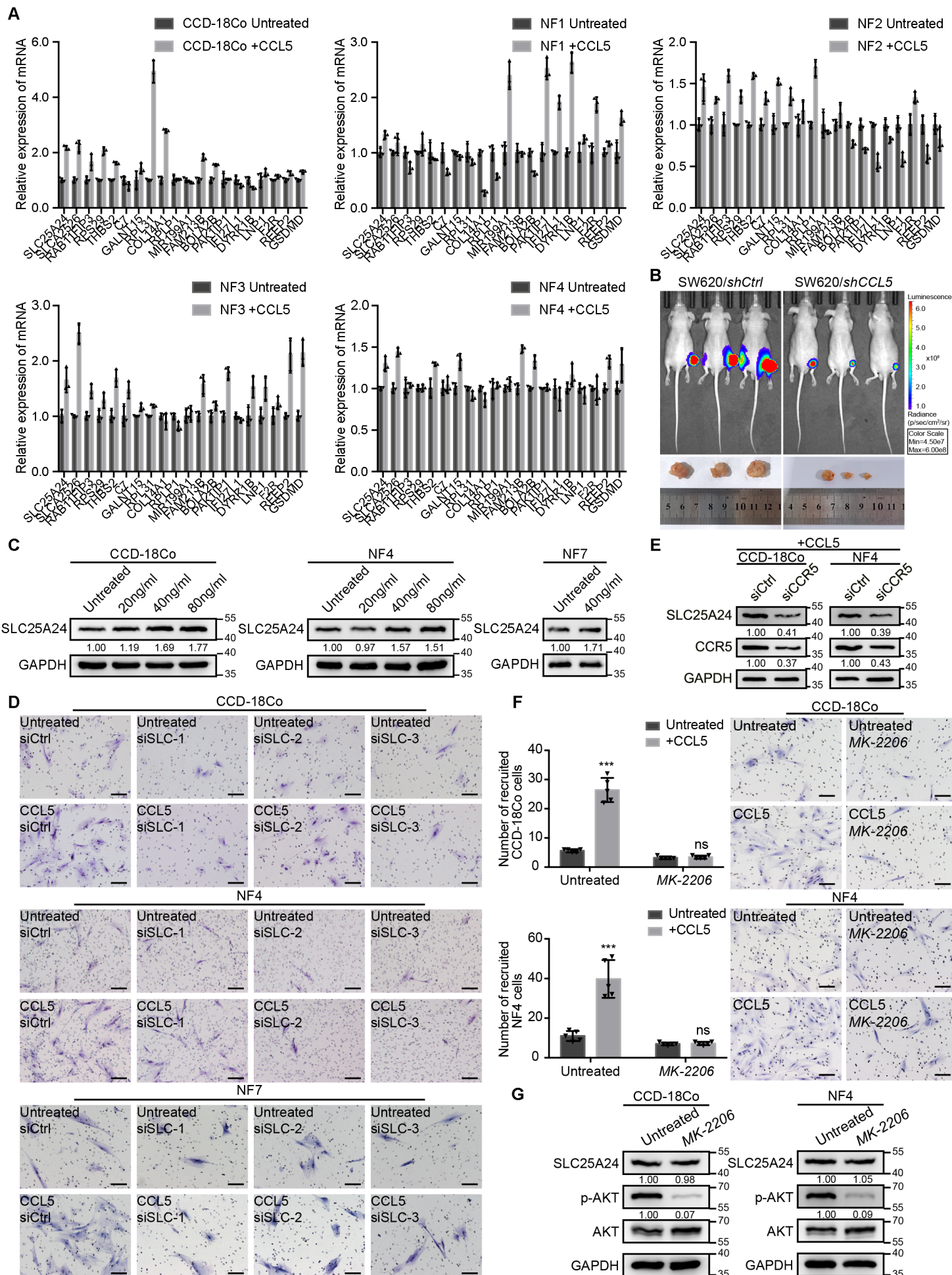

Supplement: Supplementary file 3 — Additional file 3: Figure S3. CCL5-dependent fibroblast recruitment is mediated by SLC25A24 in fibroblasts. [file 13046_2022_2300_MOESM3_ESM.pdf]

**A**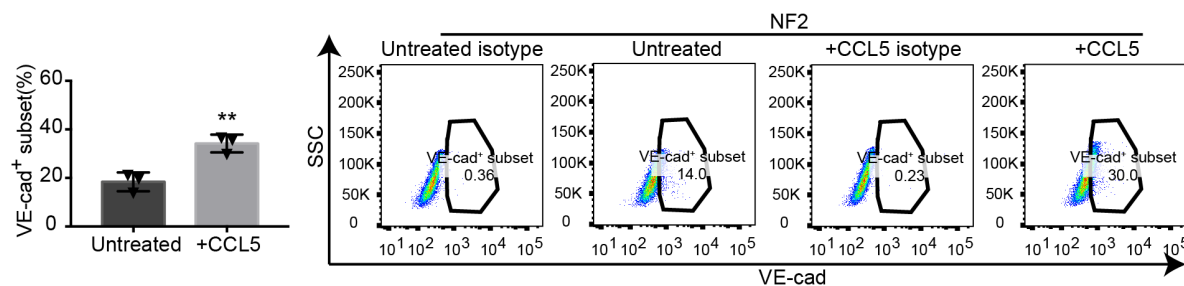**B**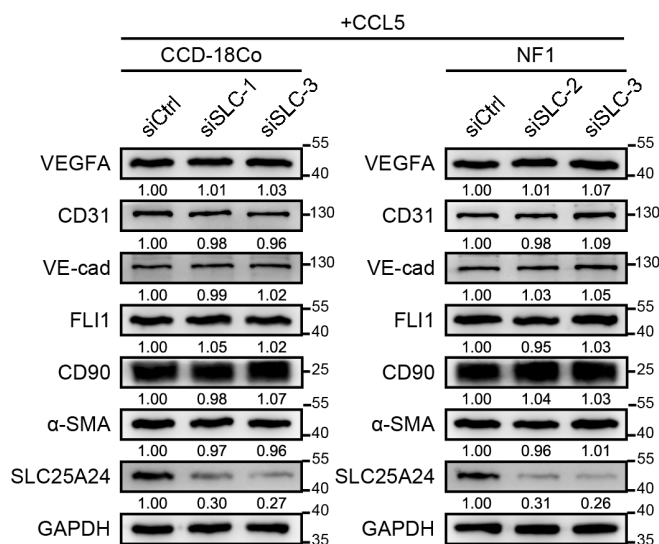**C**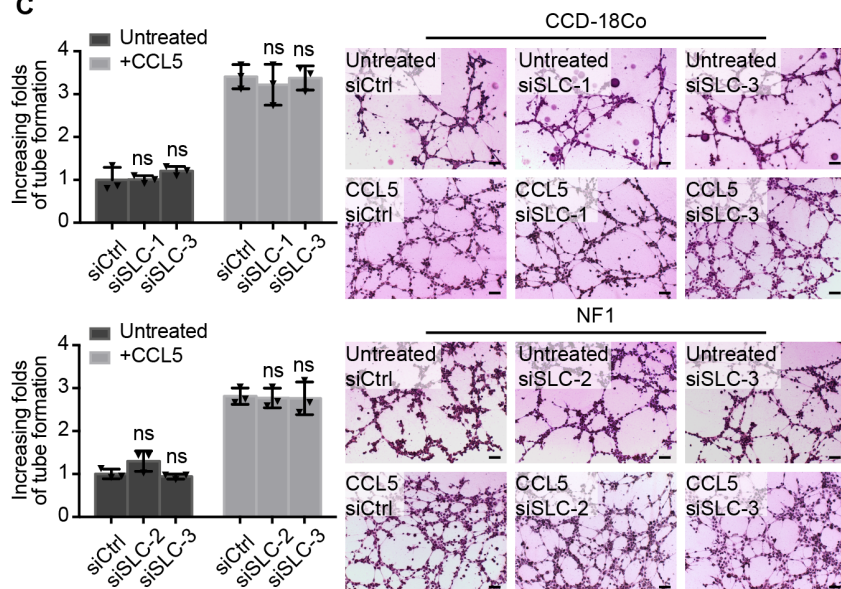

Supplement: Supplementary file 4 — Additional file 4: Figure S4. CCL5 contributes to the increase in α-SMAhigh CD90high FAPlow fibroblasts and thereby promotes tumor angiogenesis. [file 13046_2022_2300_MOESM4_ESM.pdf]

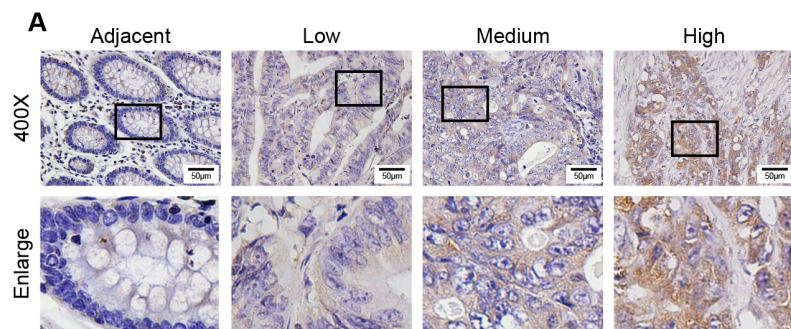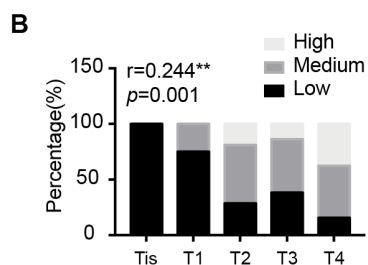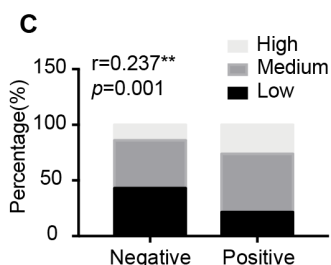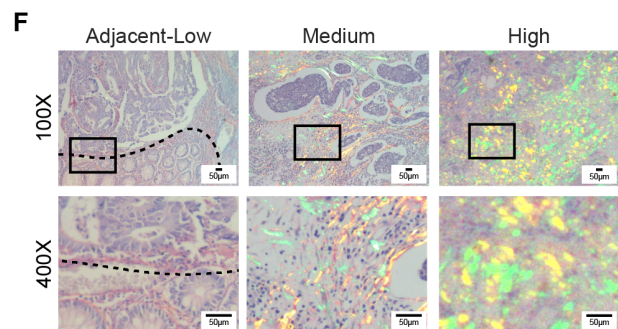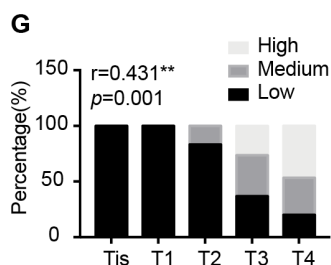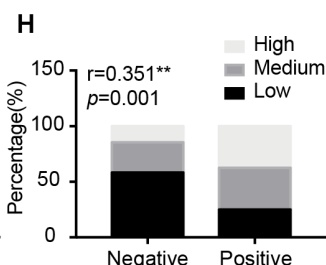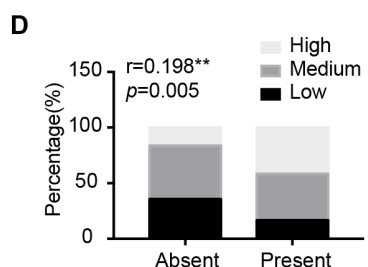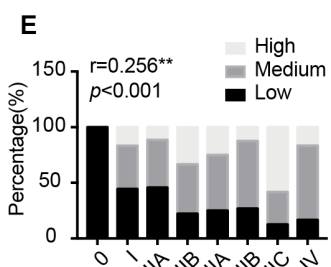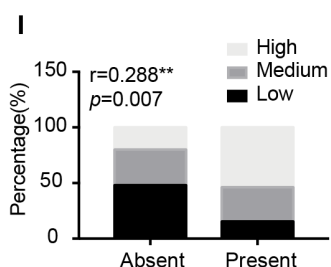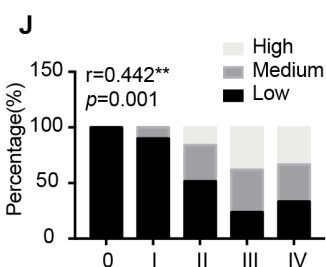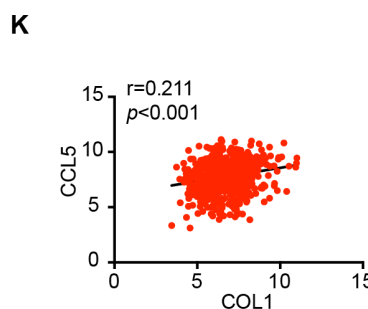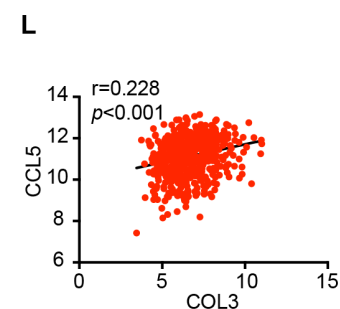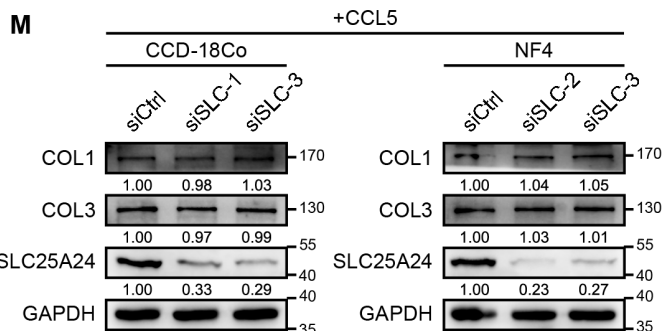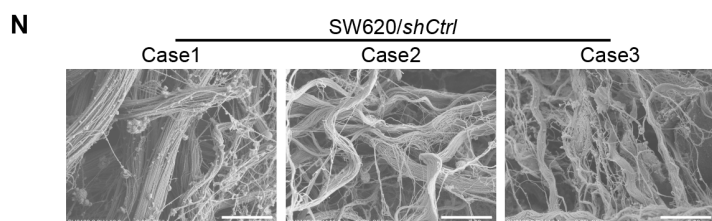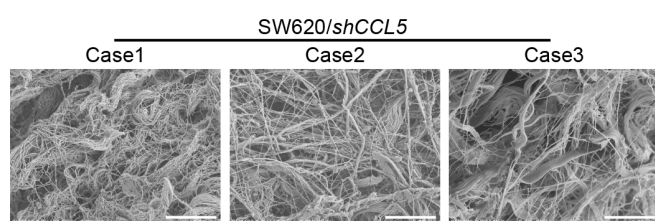

Supplement: Supplementary file 5 — Additional file 5: Figure S5. CCL5 promotes collagen synthesis via fibroblasts, contributing to tumor progression. [file 13046_2022_2300_MOESM5_ESM.pdf]
